# In vitro implementation of robust gene regulation in a synthetic biomolecular integral controller

**DOI:** 10.1038/s41467-019-13626-z

**Published:** 2019-12-17

**Authors:** Deepak K. Agrawal, Ryan Marshall, Vincent Noireaux, Eduardo D Sontag

**Affiliations:** 1 Department of Bioengineering, Northeastern University, Boston, MA USA; 2 Department of Electrical and Computer Engineering, Northeastern University, Boston, MA USA; 3 School of Physics and Astronomy, University of Minnesota, Minneapolis, MN 55455 USA; 4 Laboratory of Systems Pharmacology, Program in Therapeutic Science, Harvard Medical School, Boston MA, USA

**Keywords:** Synthetic biology, Computer modelling, Control theory, Dynamical systems

## Abstract

Feedback mechanisms play a critical role in the maintenance of cell homeostasis in the presence of disturbances and uncertainties. Motivated by the need to tune the dynamics and improve the robustness of gene circuits, biological engineers have proposed various designs that mimic natural molecular feedback control mechanisms. However, practical and predictable implementations have proved challenging because of the complexity of synthesis and analysis of complex biomolecular networks. Here, we analyze and experimentally validate a synthetic biomolecular controller executed in vitro. The controller ensures that gene expression rate tracks an externally imposed reference level, and achieves this goal even in the presence of certain kinds of disturbances. Our design relies upon an analog of the well-known principle of integral feedback in control theory. We implement the controller in an *Escherichia coli* cell-free transcription-translation system, which allows rapid prototyping and implementation. Modeling and theory guide experimental implementation with well-defined operational predictability.

## Abstract (web summary)

Feedback mechanisms for synthetic gene circuits are necessary to provide robustness to external perturbations. Here the authors validate a biomolecular controller based on a sigma and anti-sigma factor to achieve stable gene expression in the face of external disturbances in an in vitro synthetic gene circuit.

## Introduction

Robustness against perturbations and uncertainties is fundamental to biological systems that continuously sense and respond to their environment. At the cellular level, it is often desired to maintain precise control over a variety of molecular components and pathways to achieve complex behaviors that require the interaction of intracellular or extracellular biomolecules^1,2^. This is often achieved by tightly regulating gene expression in such a way that it follows a desired set point independent of exogenous or endogenous disturbances. Feedback is a mechanism that enables organisms to achieve reliable and robust functionality^3–7^. Feedback mechanisms underlie homeostasis, a phenomenon in which physiological variables are continuously monitored and adjusted so as to maintain a desired equilibrium value which is defined by a set point (also known as a reference signal), in the presence of biological uncertainties that may perturb the natural state of the system^8–10^.

Reference tracking in the presence of disturbances, a classical objective in electrical and mechanical systems, is often solved by incorporating integral feedback to control a variable of interest (also known as a plant or process signal)^11^. In such a scheme, one wants the plant signal to track a reference signal. This is achieved by incorporating in the controller the mathematical integration of the difference between the reference and the plant signal (the error signal). The internal model principle in control theory states, in essence, that the existence of an integrator in the control loop is necessary for the tight regulation of the variable of interest in the presence of constant disturbances or stimuli^12^. Inspired by these ideas from engineering and control theory, there have been several recent designs^13–16^ and implementations^17–19^ of biomolecular integral feedback controllers. While these implementations provided a general framework to robustly regulate in vivo biological processes, an alternative implementation is desirable, in order to improve the robustness of synthetic biological processes and specifically the cell-free reaction platform. In addition, the cell-free reaction platform makes the design and implementation of such a controller easier compared to in vivo systems. This is mainly due to the limitations of quantitative predictive models for the latter case. These limitations arise in large part from the biological noise which is found in cellular systems, which makes it hard to achieve precise control over model parameters, thus complicating the design and feasibility of the system^20–22^. Plasmid copy number is also limited by the origin of replication, decreasing the adjustability of experiments^23^.

In this work, we exploit the versatility of an all *Escherichia coli* TXTL platform to prototype a biological controller circuit. TXTL reactions contain the native transcription, translation, and metabolic machineries^24,25^ required to achieve gene expression over at least eight hours. As opposed to a living host, in a TXTL reaction one can precisely set the concentrations and stoichiometries of DNA parts, and thus finely tune gene circuits easily. TXTL reactions are typically performed at the microliter scale or above, far from any biological noise, thus allowing flexibility in designing and optimizing the genetic network^25^. Experimental disturbances, such as those perturbing the amount of DNA or other reaction components, can be carried out at any time. TXTL reactions are executed in high-throughput, facilitating the rapid characterization of dynamic circuits. By virtue of such advantages, several synthetic gene circuits have been implemented in TXTL with a successful modeling framework^27–30^.

In this article, we construct an in vitro synthetic biomolecular integral controller that precisely controls the protein production rate of an output gene. Our strategy relies on a molecular sequestration reaction, which is an instantiation of the innovative antithetic integral feedback paradigm introduced by Briat, Gupta, and Khammash^13^, who showed that this architecture realizes integral control and achieves robust tracking. To realize a cell-free biological controller, we exploit the natural interaction between the *E. coli* σ_28_ and the anti-σ_28_ factor^31^. Recently, a similar controller has been reported that uses the same molecular mechanism to realize integral feedback in *E. coli*^18^. In this work, we demonstrate that the output of the controller, which is realized using the TXTL toolbox, tracks the reference signal, meaning in this paper that it is linearly proportional to the input; moreover, we show that this happens for a large dynamic range of inputs. This tracking behavior is possible only in the closed-loop configuration (when the sequestration reaction is active), and not in open loop. We develop an ordinary differential equation (ODE) model and perform systematic TXTL experiments in order to parameterize and validate the model. We then use the parameterized model in order to successfully predict the controller response in different reaction conditions. When certain disturbances are added in the biochemical species as local disturbances, or in the reaction condition as a global disturbance, only the closed-loop controller enables the output to almost reject the disturbance’s effect on the output. Our results demonstrate that our synthetic biomolecular controller is capable of regulating the gene expression rate robustly in an *E. coli* TXTL toolbox. We anticipate that such an approach could be useful for diagnostics applications^32^, for constructing dynamical systems in vitro^33^ or for programming synthetic cell systems^34,35^.

## Results

### Designing an integral feedback controller

Our primary goal is to construct a genetic network which can accurately regulate the protein production rate of a target gene. Specifically, we wish to achieve reference tracking, meaning that the production rate (output) of a desired protein follows a reference signal linearly, for a large dynamic range of input values. We call tracking robust if the output tracks the reference signal even in the presence of (certain kinds of) disturbances and this behavior is maintained independently of changes in reference values over the course of the reaction.

**Fig. 1:**
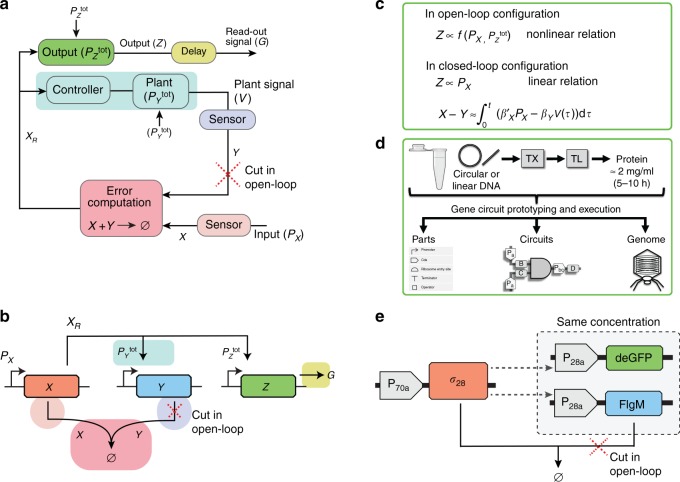
The synthetic biological integral controller. **a** Block diagram of a typical closed-loop controller. **b** Design of the synthetic biological integral controller, where the color coding corresponds to the blocks shown in **a**. The reference is set by the input DNA *P*_*X*_ and the plant signal *V* (mRNA of gene *y*) is measured through the expression of another gene *z*, which encodes for a protein *Z*. Error computation is achieved through a molecular sequestration reaction between the proteins *X* and *Y*. Here the red-color cross represents the open-loop configuration of the controller, which results when the feedback signal *Y* is absent. **c** In the closed-loop configuration, the error criterion is the difference between scaled versions of the input *P*_*X*_ and the plant signal to be regulated, *V*. In the actual controller implementation, this role is played by the free amount of the protein *X*, which we denote as *X*_*R*_ because we think of it as what remains after binding to *Y* (and to promoter sites). To produce an output (*Z*) that is independent of the disturbances in $P_Y^{{\mathrm{tot}}}$ and $P_Z^{{\mathrm{tot}}}$, the error signal is integrated (see Supplementary Note 1). The assumptions made to derive this expression are mentioned in Supplementary Note 1 where it is also shown that the controller output (*Z*) linearly depends on the plant signal at the steady state. In the absence of the feedback the output depends nonlinearly on *P*_*X*_ and $P_Z^{{\mathrm{tot}}}$ such that any disturbance in $P_Z^{{\mathrm{tot}}}$ may perturb the output. **d** Overview of the *E. coli* cell-free toolbox for prototyping and executing parts and circuits in vitro. **e** Experimental implementation of the integral controller. Three plasmids are used, P_70a_-*σ*_28_, expressing the *E. coli σ*_28_ (*X*) from a *σ*_70_ promoter (*P*_*X*_), P_28a_-FlgM, expressing the anti-*σ*_28_ factor (*Y*) from a *σ*_28_ promoter $(P_Y^{{\mathrm{tot}}})$, and P_28a_-deGFP, expressing the reporter deGFP (*G*) from a *σ*_28_ promoter $(P_Z^{{\mathrm{tot}}})$. In the open-loop controller, instead of the anti-*σ*_28_ factor (FlgM), mSA is expressed, which is not sequestered by *σ*_28_, nor does it directly affect any reaction rates (see Methods). The *mSA* control gene promoter is denoted as $P_{YC}^{{\mathrm{tot}}}$.

In electrical and mechanical control systems, integral feedback controllers are routinely used in order to achieve robust reference tracking in the presence of perturbations and uncertainties. Motivated by this analogy, various possible designs of such controllers have been discussed substantially in the context of biological systems^13–19,27^. The present work is inspired by our previous work^27^, in which we introduced a computational design based on RNA-based controllers but in which no experimental validation was provided. Here, we start from that design, modifying it to allow for direct control over genetic expression and provide an experimental validation. In this approach, the controller enables the output signal to robustly track the reference signal, which is a scaled value of an input *P*_*X*_ (Fig. 1a), and representing input gene copy number. To determine the deviation of the output signal from the reference signal, a comparison between both is required without affecting their activity. This requires that the reference and the output signals must be sensed internally using biochemical sensors. We, therefore, use a protein molecule *X*, which corresponds to the reference signal and is assumed to be proportional to the input *P*_*X*_, and a protein molecule *Y*, which corresponds to the output signal and is regulated and proportional to a plant signal *V*. This allows to realize the error computation while avoiding depletion of the reference and the plant signals. In informal terms, we may think of the concentration of free molecules *X*, which we denote as *X*_*R*_ (the *R* for remaining after binding to *Y*) as an error signal which represents the difference *X*−*Y* and influences the output to correct deviations of the output signal from the reference signal. The output block, which is not involved in the closed-loop dynamics, acts as a proxy to read the plant signal and produce an external read-out signal (see Supplementary Note 1).

Our biomolecular implementation of an integral feedback controller requires three genes: an input gene *x*, under the promoter *P*_*X*_, a target gene *y*, under the promoter $P_Y^{{\mathrm{tot}}}$ and an output gene *z*, under the promoter $P_Z^{{\mathrm{tot}}}$ (Fig. 1b). Genes *x*, *y*, and *z* are sensed using their respective encoded proteins *X*, *Y*, and *Z* via a combination of transcription and translation reactions. An error computation is achieved through a molecular sequestration between *X* and *Y*, which represent *P*_*X*_ and *V*, respectively, in such a manner that when *X* binds to *Y* or vice versa, both proteins become biologically inactive (a phenomenon, also known as annihilation)^13^. We then used the free *X* (not bound to *Y*) as a transcriptional activator that binds at the promoter regions of *y* and *z* genes. In this reaction, inactive promoters *P*_*Y*_ and *P*_*Z*_ switch to active promoters $P_Y^ +$ and $P_Z^ +$ states respectively, leading to an increased production rate of the respective encoded proteins. It is essential that the activated transcription rate from the genes *y* and *z* must be much higher than their basal expression, so that the production rate of *Y* and *Z* should be overall regulated by the error signal *X*_*R*_. Since the plant signal (*V* is a mRNA) is not a readable quantity, and *Y* gets depleted in the sequestration reactions, we use the concentration of another protein *G* (e.g., a reporter protein), which is expressed by the output gene *z*, as a read-out signal to record the controller activity. The production rate of protein *G* (considered as an output *Z*) is used as a reliable comparison metric. To ensure that the output signal truly represents the plant signal, and for that $P_Z^ +$ must truly represent $P_Y^ +$. In order to implement this, it is necessary that the same promoter must be used by the *y* and *z* genes, and the amounts of $P_Y^{{\mathrm{tot}}}$ and $P_Z^{{\mathrm{tot}}}$ should be identical^26^. Note that *x*, *y*, and *z* have the same concentrations and correspond to the biochemical species *P*_*X*_, $P_Y^{{\mathrm{tot}}}$, and $P_Z^{{\mathrm{tot}}}$, respectively.

Here and elsewhere, the term closed-loop configuration means that the feedback is present through the sequestration reaction; otherwise, when the feedback mechanism is not present, the controller is referred to as in the open-loop configuration. We establish that reference tracking is only possible in the closed-loop case, where the output linearly depends on *P*_*X*_ (Fig. 1c). In the open-loop case, the output depends nonlinearly on *P*_*X*_ and $P_Z^{{\mathrm{tot}}}$, which means that, according to our definition, the open-loop system is unable to track the reference signal. Because the error signal (*X*_*R*_) is mathematically integrated over time in the closed-loop case, the output should be able to track the reference signal even when appropriately placed disturbances are added to the genetic network (Fig. 1a, c).

For a case when the reference signal is larger than the output signal, the error signal increases the production of the output *Z*. On the other hand, when the output signal is larger than the reference signal, free *Y* sequesters *X* to reduce the output. One of the key reasons that this controller is able to improve substantially upon that in ref. ^27^ is that it implements an effective error computation through protein interactions; in contrast, RNA-based designs suffer from the fact that RNAs degrade much faster than proteins, making an effective error computation very hard to implement experimentally^36^. In contrast, the degradation of the proteins used in this work can be ignored as no degradation tags were associated with them. However, while realizing the integral controller in vivo, the adverse effect of degradation on reference tracking due to cell dilution should be considered^21^.

To test the controller experimentally in *E. coli* TXTL toolbox (Fig. 1d), we employed three plasmids (Fig. 1e): P_70a_-*σ*_28_, expressing *E. coli σ*_28_ from a *σ*_70_ promoter; P_28a_-FlgM, expressing the anti-*σ*_28_ factor (also known as FlgM) from a *σ*_28_ promoter; and P_28a_-deGFP, expressing the reporter deGFP from a *σ*_28_ promoter. In the open-loop controller, gene expressing FlgM is replaced by a gene expressing mSA (same protein size), which cannot sequester with *σ*_28_, nor does it directly affect any reaction rates (see Methods). Here and elsewhere, for simplification, *σ*_28_, anti-*σ*_28_ factor (FlgM), and immature deGFP are denoted as *X*, *Y*, and *Z*, while promoters P_70a_ and P_28a_ are denoted as *P*_*X*_ and $P_Y^{{\mathrm{tot}}}$ (same as $P_Z^{{\mathrm{tot}}}$), respectively, and the *mSA* control gene is denoted as *yc* and the respective promoter as $P_{YC}^{{\mathrm{tot}}}$.

### The closed-loop controller tracks the reference signal

**Fig. 2:**
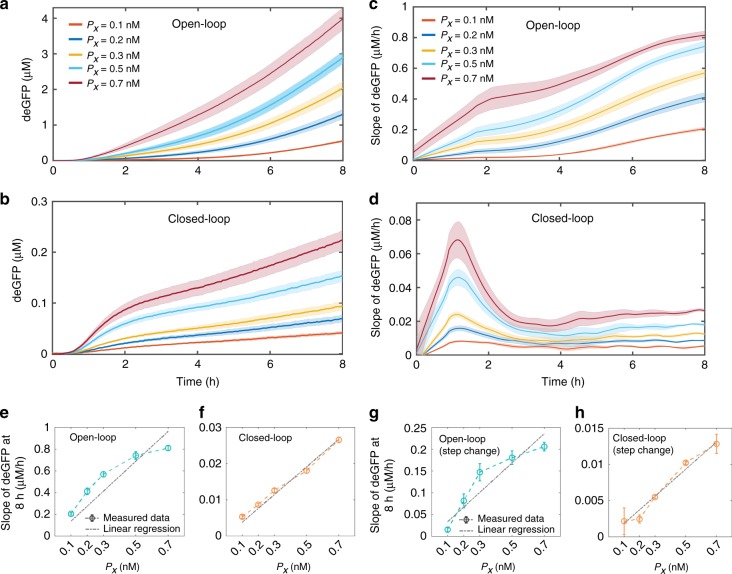
The output follows the input signal linearly only in the closed-loop configuration. **a, b** TXTL deGFP measurement of the response of the integral controller in the **a** open-loop and **b** closed-loop configurations at different initial concentrations of *P*_*X*_ (0.1–0.7 nM) while initial $P_Y^{{\mathrm{tot}}}$ and $P_Z^{{\mathrm{tot}}}$ were both 1 nM each. In the open-loop, instead of $P_Y^{{\mathrm{tot}}}$, $P_{YC}^{{\mathrm{tot}}}$ was added. **c, d** The slopes of measured deGFP responses for the **c** open-loop and **d** closed-loop operations and the corresponding summary in **e** and **f** at 8 h respectively. To disable the feedback in the open-loop case $P_Y^{{\mathrm{tot}}}$ was replaced by $P_{YC}^{{\mathrm{tot}}}$, which expresses a protein that cannot sequester with *X*. **g, h** Summary of the deGFP slopes of the controller at 8 h for a step change in *P*_*X*_ for the **g** open-loop and **h** closed-loop operations. *P*_*X*_ was increased from 0 nM to different concentrations (0.1–0.7 nM) after 4 h of the reaction in the presence of initial 1 nM *of* $P_Y^{{\mathrm{tot}}}$ and $P_Z^{{\mathrm{tot}}}$ each. Note that the lower GFP slope values in **g, h** than **e, f** are due to the shorter active reaction time. Error are shown in the shaded region and were determined using the standard error of the mean of three or more repeats. A linear regression with zero intercept was used to fit the deGFP slopes and the corresponding *R*-square values are **e** 0.71, **f** 0.98, **g** 0.84, and **h** 0.98. A calibration factor was used to convert the measured deGFP fluorescent signal into the concentration. Before calculating deGFP slopes, measured deGFP responses were smoothed-out using the *rloess* smoothing method in MATLAB. Source data are provided as a Source Data file.

Our first goal was to establish that when the controller operates in the closed-loop configuration, the output of the controller (*Z*) follows linearly the changes in the concentration of input *P*_*X*_, thus tracking the reference signal. A nonlinear dependence of the output on *P*_*X*_ would suggest otherwise. To test this in TXTL reactions, we added 0.1–0.7 nM *P*_*X*_ and 1 nM each of $P_Z^{{\mathrm{tot}}}$ and either $P_{YC}^{{\mathrm{tot}}}$for the open-loop operation (Fig. 2a) or $P_Y^{{\mathrm{tot}}}$ for the closed-loop operation (Fig. 2b). In our implementation, the controller’s output is an immature version of the reporter protein deGFP, and its matured version, which is fluorescent, is considered as a read-out signal (*G*). Since the fluorescent deGFP has no degradation tag; consequently, we cannot observe a steady-state behavior in the measured responses. Therefore, to be able to compare the open and closed-loop responses accurately, we determined the slopes of the measured deGFP signals (Fig. 2c, d), which is also referred to as the production rate of deGFP. It can be shown that deGFP slope is a scaled version of *Z*, which follows the plant signal linearly at the steady-state in the closed-loop configuration (Supplementary Note 1). In the open-loop case, we found that changes in the deGFP slopes depend nonlinearly on the changes in the input concentration of *P*_*X*_ (Fig. 2e), suggesting that the output is unable to track the reference signal over the tested range. In contrast, the closed-loop endpoint deGFP slopes were linearly proportional to the concentration of *P*_*X*_ (Fig. 2f), suggesting reference tracking.

The output of the closed-loop controller should be able to follow the changes in input signal linearly independent of the time when it is modified. To test this capability, we performed a step change in the input *P*_*X*_ concentration during the course of the reaction. For that, we added different amounts of *P*_*X*_ to TXTL reactions in the open-loop (Supplementary Fig. 1a, b) and closed-loop (Supplementary Fig. 1c, d) system after four hours of incubation with 1 nM each of $P_Z^{{\mathrm{tot}}}$ and either $P_{YC}^{{\mathrm{tot}}}$ or $P_Y^{{\mathrm{tot}}}$, respectively. As mentioned earlier, we observed that the controller’s output follows linearly the input only when operated in the closed-loop configuration (Fig. 2g, h). Note that the deGFP produced in the closed-loop configuration is much smaller than that produced in the open-loop configuration because the activator needed to express the deGFP is sequestered only in the closed-loop configuration.

The open-loop controller output is unable to track the input signal. One possibility is that this observation can be explained by the higher output levels in the open-loop case. To eliminate this possibility, we tested the controller operation in a different reaction condition, by ensuring that the open-loop output levels are in close proximity to the closed-loop output levels (shown in Fig. 2b) at the same input *P*_*X*_ values. We found that the open-loop controller output remains nonlinear as a function of *P*_*X*_, even when the open and closed-loop output levels are similar (Supplementary Fig. 2; Fig. 2f). This provides a controlled comparison and shows that feedback is responsible for the reference tracking behavior. As a control, we also tested that changes in the concentration of $P_{YC}^{{\mathrm{tot}}}$ have no effect on the deGFP slopes (Supplementary Fig. 3), confirming that the different version of *Y* that is expressed by *yc* gene does not interact with *X*. In contrast, when we increased the concentration of $P_Y^{{\mathrm{tot}}}$, from 0 to 1 nM in the presence of *P*_*X*_ and *P*_*Z*_, the deGFP slopes reduced significantly, suggesting that the sequestration reaction is being actively involved in regulating the error signal and thereby the output activity (Supplementary Fig. 4). We also found that in the absence of *P*_*X*_, deGFP is not produced (Supplementary Fig. 5), confirming that the production of *Y* and *Z* are fully governed by the error signal through the activation reaction.

These experimental observations agree with the expected controller operation. When *X* is larger than *Y* (i.e., the output is lagging behind the reference). The error signal increases the production of *Y* and *Z*. As more *Y* is available in the reaction to sequester with *X*, *X* and *Y* converge to specific values that would allow *Z* to follow the reference signal. In the absence of the sequestration reaction, error computation is absent (no feedback) and so *X* directly regulates *Z* production without comparing with the reference signal.

### Mathematical model and parameterization

**Fig. 3:**
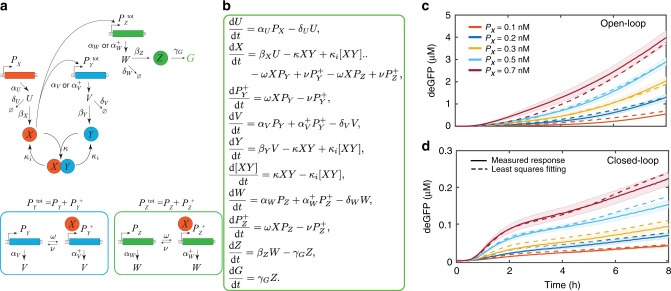
Schematic of the model parametrization process and validation of the model. **a** Detailed reaction network of the controller and the corresponding. **b** ODE model. Here *U*, *V,* and *W* are the translationally initiated mRNA of the *X*, *Y*, and *Z* proteins, respectively. The additional reaction was added into the model to consider the maturation of immature deGFP into a fluorescent deGFP (*G*). Details on how the $P_Y^{{\mathrm{tot}}}$ and $P_Z^{{\mathrm{tot}}}$ promoters switch from the inactive (*P*_*Y*_, *P*_*Z*_) to active $(P_Y^ + ,P_Z^ + )$ states are shown alongside. Note that while using the ODE model to study the controller dynamics, *P*_*Y*_ and *P*_*Z*_ were replaced with $P_Y^{\mathrm{tot}} - P_Y^ +$ and $P_Z^{\mathrm{tot}} - P_Z^ +$ respectively. **c, d** Comparing the measured deGFP response (solid lines) with the mean of the best-fitted simulation results (dashed lines) for the **c** open-loop and **d** closed-loop cases. The ODE model was used to calculate the controller response while initial concentrations of $P_Y^{{\mathrm{tot}}}$ and $P_Z^{{\mathrm{tot}}}$ were 1 nM each. Note for the open-loop response *α*_*V*,_ *α*_*V*_^*+*^, *δ*_*V*,_ *β*_*Y*_, *κ*, *κ*_i_, and $P_Y^{{\mathrm{tot}}}$ were set to zero. Least squares fitting was used to generate the best-fit model response at different initial guesses of the reaction parameters (see Methods). Experimentally observed error bars are shown in the shaded region while the mean simulated trajectories (dashed line) are shown here within 95% confidence intervals. *N* = 1000. Error bars are from the SEM of at least three repeats. Source data are provided as a Source Data file.

To understand the controller operation, we developed a simple coarse-grained model that captures the dynamic response of the controller in both open and closed-loop configurations (Fig. 3a). For that, we consider the synthesis of each protein as a two-step reaction: a transcription reaction for mRNA synthesis and then a translation reaction for the corresponding protein synthesis. The parameters *α* and *β* are transcription and translation rates, respectively. Here and elsewhere, subscripts to the parameters indicate the corresponding species. Each mRNA species (*U*, *V*, and *W*) has a degradation rate denoted as *δ* while we ignore the protein degradation rate^36^. The parameter *κ* is the sequestration rate. Transcriptional activation is modeled as a one-step reaction, where *X* binds to the promoters *P*_*Y*_ and *P*_*Z*_ separately at a rate of *ω* and dissociates at a rate of *ν*. In the activated state, these genes produce *Y* and *Z* proteins at an increased transcription rate, denoted as *α*^*+*^. Considering the mass-conservation, we assume that $P_Y^{{\mathrm{tot}}} = P_Y + P_Y^ + \,{\mathrm{and}}\,P_Z^{{\mathrm{tot}}} = P_Z + P_Z^ +$ at all times. An additional reaction is added into the model to account for the maturation of newly synthesized immature deGFP (*Z*) into a fluorescent deGFP (*G*)^25,33^, which is the read-out signal (Fig. 3a). We have also added a reverse sequestration reaction that allow sequestered *X* and *Y* to dissociate at a rate denoted as *κ*_i_. From chemical reactions, we built an ODE model (shown in Fig. 3b) to determine the response of the controller over time.

An accurate representation of a system model requires determining specific parameter values at which the model quantitatively follows the system dynamics. However, parameter estimation can be nontrivial, as there can be multiple sets of parameter values that may vary by several orders of magnitude and could still fit the measured data. Therefore, to simplify the problem, we isolated the measured responses into two sets, in such a manner that we required fewer parameters to fit a particular set of experimental data. For this, we first found the model parameter values that provided the best fit to the measured open-loop response, since the number of parameters involved in the open-loop case are less than in the closed-loop case (see Methods). We then used these parameter values to fit the closed-loop response while allowing only the remaining parameters to vary (see Methods). Moreover, we started the model fitting manually with an initial guess of parameters derived from the literature^24–30,36^. Once we found the possible values that provide a qualitative agreement between the model and the measured response, we used an iterative least-squares fitting procedure to find a range of parameter values that gave us the best fit (see Methods). The means of the resulting parameters (Table 1) were then used along with the ODE model (Fig. 3b) to calculate the mean trajectories with 95% confidence intervals (Fig. 3c, d). A comparison of model response with the deGFP slopes is shown in Supplementary Fig. 6. Histograms of the input and estimated parameter distributions are shown in Supplementary Fig. 7.

**Table 1:** Estimated model parameters obtained from the least squares fitting for the ODE model shown in Fig. 3b.

Parameters	Values		Error	Units
*α*_*U*_	0.60	±	0.01	s^−1^
*α*_*V*_	2.75 × 10^−5^	±	8.35 × 10^−7^	s^−1^
*α*_*V*_^*+*^	0.51	±	0.0123	s^−1^
*α*_*W*_	3.68 × 10^−7^	±	9.76 × 10^−9^	s^−1^
*α*_*W*_^*+*^	0.78	±	0.0013	s^−1^
*δ*_*U*_	0.00501	±	7.23 × 10^−5^	s^−1^
*δ*_*V*_	0.00026	±	2.57 × 10^−6^	s^−1^
*δ*_*W*_	0.00109	±	3.05 × 10^−6^	s^−1^
*β*_*X*_	0.00046	±	1.41 × 10^−5^	s^−1^
*β*_*Y*_	0.0018	±	4.10 × 10^−5^	s^−1^
*β*_*Z*_	0.00098	±	1.53 × 10^−6^	s^−1^
*κ*	8.99 × 10^4^	±	8.88 × 10^2^	M^−1^ s^−1^
*κ*_i_	1.0 × 10^−4^	±	1.42 × 10^−6^	s^−1^
*ω*	8.02 × 10^5^	±	7.34 × 10^3^	M^−1^ s^−1^
*ν*	1.54	±	0.07	s^−1^
*ϒ*_*G*_	1.95 × 10^−3^	±	4.86 × 10^−6^	s^−1^

Note for the open-loop response *α*_*V*,_ *α*_*V*_^*+*^, *δ*_*V*,_ *β*_*Y*_, *κ*, *κ*_i_, and *P*_*Y*_^tot^ were set to zero. The error values were determined using the standard error of the mean. *N* = 1000

**Fig. 4:**
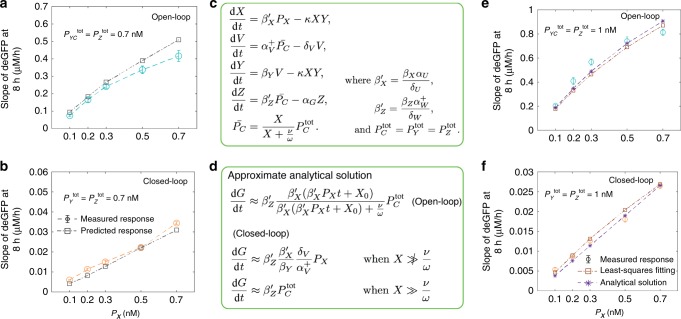
Validation of the controller model and simplification. **a, b** Predicting the **a** open-loop and **b** closed-loop controller response for a step change in *P*_*X*_. *P*_*X*_ was increased from 0 nM to different concentrations (0.1–0.7 nM) after 2 h of incubation in the presence of initial 0.7 nM of $P_Y^{{\mathrm{tot}}}$ and $P_Z^{{\mathrm{tot}}}$ each. (Recall that fitting was done under different conditions, namely 1 nM of *P*_*YC*_^tot^ (or $P_{Y}^{\mathrm{tot}}$) and $P_{Z}^{\mathrm{tot}}$ each.) The ODE model shown in Fig. 3b was used to determine the response with parameters shown in Table 1. **c** Simplified ODE model of the controller. **d** Approximate analytical solution for deGFP slopes (time derivative of *G*) for the open and closed-loop cases. **e**, **f** Comparing the measured responses of the controller shown in Fig. 3c, d with the response determined using the approximate analytical solution for the **e** open-loop and **f** closed-loop cases respectively. The data shown in Fig. 4a, b are compared with the analytical solution results in Supplementary Fig. 15. Before calculating deGFP slopes, measured deGFP responses were smoothed-out using the *rloess* smoothing method in MATLAB. Error bars are from the SEM of at least three repeats. Source data are provided as a Source Data file.

To cross-validate the parameterized model, we predicted the controller response for a different setting of input conditions. For that, the concentration of *P*_*X*_ was increased from 0 nM to 0.1–0.7 nM (in a step manner) after 2 h of incubation in the presence of initial 0.7 nM of $P_Y^{{\mathrm{tot}}}$ and $P_Z^{{\mathrm{tot}}}$ each (Fig. 4a, b; Supplementary Fig. 8). We found that the predicted responses followed the measured responses closely. It should be noted that the reason, in this study, we have limited to model the measured dynamic trajectories for up to 8 h. This is because experimentally, after 8 h, we observed the effects of resource limitations (Supplementary Fig. 9) and the ODE model shown in Fig. 3b, assumes an unlimited energy source and therefore ignores the experimental results when that assumption no longer holds.

### Model simplification

To get a better insight into the controller response, further simplification of the proposed model is required. For that, based on the extracted parameter values, we sought to reduce the number of variables in the model while still capturing the essential dynamics of the controller. From the extracted parameter values, we observed that the basal expression of *y* and *z* genes is almost negligible; therefore, we set *α*_*V*_ and *α*_*W*_ to zero in the simplified model. As the transcriptional activation reaction is much faster than the other reactions involved in the reaction network, we used a quasi-steady state approximation to replace the ODEs of $P_Y^ +$ and $P_Z^ +$ by their steady-state expressions (see Supplementary Note 1). As the RNA dynamics is much faster than the protein dynamics (due to the faster RNA degradation rates), a similar approximation was used to model the synthesis of *X* and *Z* using single reactions for each, while keeping the two-step synthesis of *Y* to ensure that we consider an appropriate delay in the overall system dynamics (Supplementary Fig. 10)^37^. We also ignored the reverse sequestration reaction (*κ*_i_) assuming it has a limited impact on the dynamics of the deGFP (Supplementary Fig. 11). This leads to a simplified model (shown in Fig. 4c), which can produce a dynamic response similar to that of the original model (Supplementary Fig. 12).

We now use the simplified model to determine analytically how the output depends on the reaction parameters and the input. As mentioned earlier, because the reporter protein deGFP (*G*) has no degradation tag, we opted to analyze the slope of deGFP (scaled value of *Z*), and a steady-state response can be seen for the closed-loop controller (Fig. 2d). However, in the given implementation, it is not possible to observe a steady-state response of *Z* in the open-loop configuration (Fig. 2c). This is because as the sequestration reaction is absent in the open-loop case, *X* continues to accumulate over time and so does *Z* (Supplementary Fig. 13; Fig. 2c). In contrast, due to the sequestration reaction in the closed-loop operation, *X* attains a steady-state, which makes *Z* to attain a steady-state as well (Supplementary Fig. 14; Fig. 2d).

To determine an approximate analytical expression for the slope of deGFP, we used the simplified model to obtain an approximate analytical solution of *Z* (see Supplementary Note 1). We then analytically calculated the time derivative of *G* for the open and closed-loop cases (Fig. 4d). The analytical solution follows the response of the original model closely at different reaction conditions (Fig. 4e, f; Supplementary Fig. 15), and thus, validating our approach. Moreover, we found that the time derivative of *G* (same as slope of deGFP) follows the input *P*_*X*_ linearly only in the closed-loop case as long as there are enough promoter sites are available on *y* and *z* genes. This is due to the fact that in the closed-loop configuration, *Z* is independent of the promoter activity of *z* gene as long as *X* ≫ *ω/ν* is not valid. This is considered as a fundamental limit of the controller to track the reference signal robustly. In contrast, in the open-loop case, as the sequestration reaction is absent, production of *Z* directly depends on the promoter activity of *z* gene which causes a nonlinear dependency of *Z* on *X*, thereby on *P*_*X*_ (Fig. 4d). In the closed-loop case when *X* ≫ *ω/ν*, *Z* converges to a saturation. We verified these conclusions using the numerical simulations (see Supplementary Fig. 16). Moreover, the closed-loop controller capability to track the reference signal should be independent of the absolute value of the output when *X* ≫ *ω/ν* is not valid. To test this, we conducted numerical simulations at different initial DNA concentrations values for the open and closed-loop cases in such a manner that the controller’s output remains in the proximity in these two cases. We observed linear relation between the input *P*_*X*_ and the output only for the closed-loop case (Supplementary Fig. 17).

Further insight into the controller operation can be gained by analyzing the simplified model to determine how the error signal is processed in the closed-loop configuration of the controller. The analytical expression for the error signal clearly shows that it is integrated mathematically by the controller (see Supplementary Note 1). We then further validate the conditions under which *Z* faithfully tracks the plant signal, which ensures accurate reading of the controller dynamics (see Supplementary Note 1).

### Closed-loop control enables disturbance rejection

One of the main theoretical advantages of integral feedback controllers is their ability to reduce the effect on the output of certain constant disturbances on the variable of interest^9^. This is due to the effective error computation with an integral operation that allows the controller to maintain the desired output even when a disturbance affects the system. In the aforementioned section, we showed that the implemented controller can be interpreted as an integral feedback mechanism (see Supplementary Note 1). Therefore, we expect that the closed-loop system is able to suppress disturbances in an appropriate sense. To test this, we introduced disturbances in the concentration of the biochemical species $P_Y^{{\mathrm{tot}}}$ and $P_Z^{{\mathrm{tot}}}$. In practical settings, variation in the DNA concentration is one of the most biologically relevant parameters. This is because in vivo gene concentration can vary significantly due to fluctuations in plasmid copy number^38^, and several designs have been proposed in order to ameliorate the effect of copy number variation^39,40^. Notably, the TXTL reaction platform allows us to design such an experiment, where DNA template concentrations can be changed at any time during the course of a reaction due to the TXTL reaction settings.

In this design, the steady-state value of the closed-loop controller’s output is independent of the amount of *y* and *z* genes (in Fig. 4d), and this implies that any disturbances in these species should not perturb the deGFP response when the controller is operated only in the closed-loop configuration. To test this, we first used the ODE model (Fig. 3b) to predict the controller response in the open-loop and closed-loop configurations where the concentrations of $P_Y^{{\mathrm{tot}}}$ and $P_Z^{{\mathrm{tot}}}$ were varied from 0.2 to 0.7 nM, keeping a fixed 0.2 nM initial *P*_*X*_. We observed a less than ~1.2-fold variation in the deGFP slopes for the closed-loop case, compared to ~4-fold variation in the open-loop case (Supplementary Fig. 18). To understand these further, we conducted numerical simulations and analyzed the dynamics of each species involved in the genetic network. We found that in the closed-loop operation, an increase in $P_Z^{{\mathrm{tot}}}$ increases the amount of *Y*, but as more *Y* is available to sequester *X*, less free *X* (not bound to *Y*) is available to activate the production of *Y* and *Z*. Even though $P_Z^{{\mathrm{tot}}}$ was increased, free *X* is reduced such that the reporter protein *G* (and also the output *Z*) remains almost the same, independently of the amount of $P_Z^{{\mathrm{tot}}}$ (Supplementary Fig. 19). For the open-loop case, as there is no feedback, an increase in $P_Z^{{\mathrm{tot}}}$ significantly increases the production rate of *G* (Supplementary Fig. 20).

**Fig. 5:**
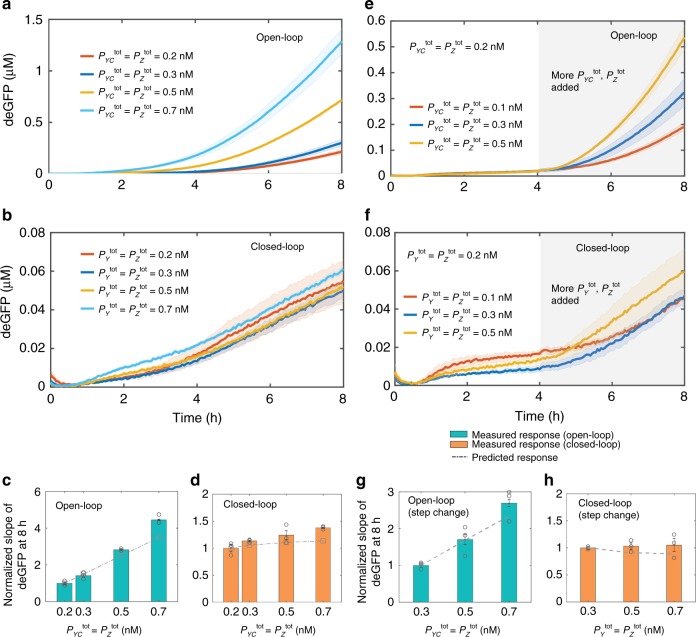
Closed-loop operation enables the controller to achieve robustness to the local disturbance on the system. **a, b** Measured deGFP response of the controller in the presence of disturbances in the concentration of *P*_*YC*_^tot^ (or $P_{Y}^{{\mathrm{tot}}}$) and $P_Z^{{\mathrm{tot}}}$ (0.2–0.7 nM) for the **a** open-loop and **b** closed-loop cases while initial *P*_*X*_ was 0.2 nM. The error bars are shown in the shaded region and were determined using the standard error of the mean of three or more repeats. **c**, **d** Summary of the deGFP slopes of the controller at 8 h for the **c** open-loop and **d** closed-loop operations. Error bars are from the SEM of at least three repeats. To disable the feedback in the open-loop case $P_Y^{{\mathrm{tot}}}$ was replaced by $P_{YC}^{{\mathrm{tot}}}$, which expresses a protein that cannot sequester with *X*. **e, f** Measured response of the controller when the disturbance in $P_Y^{{\mathrm{tot}}}$ and $P_Z^{{\mathrm{tot}}}$ was added in a step manner for the **e** open-loop and **f** closed-loop cases. Additional $P_Y^{{\mathrm{tot}}}$ and $P_Z^{{\mathrm{tot}}}$ were added (0.1–0.5 nM) after 4 h of the reaction in the presence of initial 0.2 nM of *P*_*X*_, $P_Y^{{\mathrm{tot}}}$ and $P_Z^{{\mathrm{tot}}}$ each. **g**, **h** Summary of the normalized deGFP slopes of the controller at 8 h for the **g** open-loop and **h** closed-loop operations. Normalization was done with respect to the first slope value for each variation in $P_Y^{{\mathrm{tot}}}$ and $P_Z^{{\mathrm{tot}}}$. The predicted response for each case was determined using the ODE model shown in Fig. 3b with parameters shown in Table 1. Before calculating deGFP slopes, measured deGFP responses were smoothed-out using the *rloess* smoothing method in MATLAB. Source data are provided as a Source Data file.

Encouraged by these results, we experimentally tested the same conditions in TXTL reactions. We added 0.2 nM *P*_*X*_ and increasing concentrations of $P_Y^{{\mathrm{tot}}} = P_Z^{{\mathrm{tot}}}$, from 0.2 nM to 0.7 nM to reactions, and tracked the deGFP signal over time for the open-loop (Fig. 5a) and the closed-loop (Fig. 5b) configurations. In the open-loop case, the output signal increased by more than ~4.5-fold with the increasing concentration of $P_{Yc}^{{\mathrm{tot}}} = P_Z^{{\mathrm{tot}}}$ due to the lack of feedback (Fig. 5c). However, in the closed-loop case, the output signal was increased by only ~1.4-fold with increasing $P_Y^{{\mathrm{tot}}} = P_Z^{{\mathrm{tot}}}$, thus confirming that the controller suppressed the disturbance in their concentrations (Fig. 5d), as predicted by integral feedback theory.

Similarly to reference tracking, rejection of disturbances should be independent of the time when they are introduced in the system. To test this, we characterized the controller response to a step change in the concentrations of $P_Y^{{\mathrm{tot}}}$ and $P_Z^{{\mathrm{tot}}}$ as disturbances. We started the reaction with *P*_*X*_, $P_{YC}^{{\mathrm{tot}}}$ and $P_Z^{{\mathrm{tot}}}$ concentrations each set to 0.2 nM, and after 4 h of incubation, additional $P_{YC}^{{\mathrm{tot}}}$ and $P_Z^{{\mathrm{tot}}}$ were added (see Methods) (Fig. 5e). For the closed-loop case, instead of $P_{YC}^{{\mathrm{tot}}}$, $P_Y^{{\mathrm{tot}}}$ was added in the same amounts (Fig. 5f). The output was changed by less than ~1.1-fold in the presence of the disturbances only in the closed-loop case, as expected based on our understanding of the controller compared to ~2.7-fold change in the open-loop case (Fig. 5g, h). It should be noted that cases where the disturbances were added after 4 h of the reaction (Fig. 5g, h), a lower deGFP expression results, due to the limited amount of $P_Y^{{\mathrm{tot}}}$ and $P_Z^{{\mathrm{tot}}}$ compared to when the disturbances were added at the beginning of the reaction (Fig. 5c, d) for the same concentration of *P*_*X*_, $P_Y^{{\mathrm{tot}}}$ and $P_Z^{{\mathrm{tot}}}$. We also tested the controller’s response for a wide range of disturbances when different initial concentrations of DNA were used, and the disturbance was added in different amounts (see Supplementary Figs. 21 and 22). In each case, we found a similar robust controller response in the closed-loop settings. Moreover, the open-loop analytical expression (Fig. 4d) suggested that the open-loop controller cannot suppress the disturbances independently of the absolute value of the output. We, therefore, tested the open-loop controller operation at a lower initial *P*_*X*_ value to produce an output response that is much lower than the closed-loop response shown in Fig. 5a while adding the same amount of disturbance in $P_{YC}^{{\mathrm{tot}}}$ and $P_Z^{{\mathrm{tot}}}$. We observed that the open-loop output level was increased by ~4-fold with the increase in DNA concentration and thus further validating the mathematical model of the controller (see Supplementary Fig. 23).

**Fig. 6:**
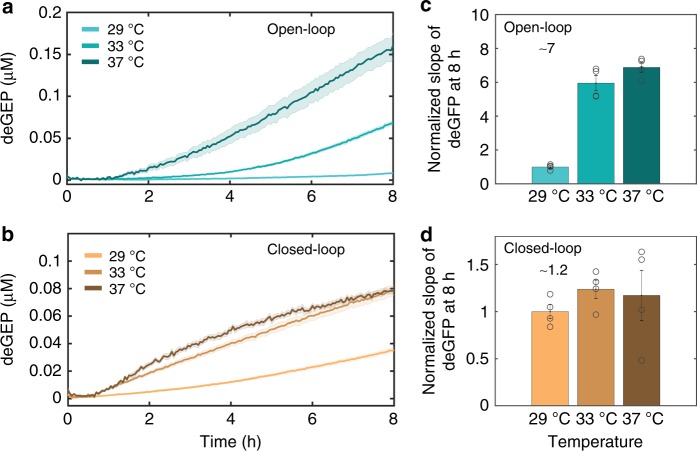
Closed-loop controller enables robustness to the global perturbation. **a**, **b** Measured response of the controller at three different external constant change in the reaction temperatures for the **a** open-loop ($P_{YC}^{{\mathrm{tot}}}$ and $P_Z^{{\mathrm{tot}}}$ were both 0.1 nM) and **b** closed-loop ($P_Y^{{\mathrm{tot}}}$ and $P_Z^{{\mathrm{tot}}}$ were both 1 nM each) cases while initial *P*_*X*_ was 0.1 nM. The error bars are shown in the shaded region and were determined using the standard error of the mean of three or more repeats. **c**, **d** Summary of the normalized deGFP slopes of the controller at 8 h for the **c** open-loop and **d** closed-loop operations. Error bars are from the SEM of at least three repeats. To disable the feedback in the open-loop case, $P_Y^{{\mathrm{tot}}}$ was replaced by $P_{YC}^{{\mathrm{tot}}}$, which expresses a protein that cannot sequester with *X*. The responses shown in **c**, **d** were normalized with respect to the deGFP slope value recorded at 29 °C. Plate readers were calibrated at 29 °C, 33 °C, and 37 °C separately to a standard curve of GFP to ensure fluorescence variation reflects protein concentration variation (see Supplementary Fig. 24). Before calculating deGFP slopes, measured deGFP responses were smoothed-out using the *rloess* smoothing method in MATLAB. Source data are provided as a Source Data file.

Finally, we tested the operation of the controller subject to a global perturbation, such as a variation in a specific environmental condition that potentially affects several parameters simultaneously. One of such most natural global perturbations is variation in temperature. We, therefore, carried an experiment where the controller response was recorded at three different temperatures (29 °C, 33 °C, and 37 °C) in the open and closed-loop configurations (Fig. 6; Supplementary Fig. 25). To obtain similar output levels in the open-loop case as in the closed-loop case, different initial values of $P_Z^{{\mathrm{tot}}}$ (same as $P_{YC}^{{\mathrm{tot}}}$ and $P_Y^{{\mathrm{tot}}}$ for open-loop and closed-loop cases, respectively) were used while keeping the same input *P*_*X*_ values. We found that our controller is strikingly capable of suppressing the global disturbance caused by the change in the reaction temperature: while the open-loop system leads to a ~7-fold change in response, the closed-loop system only has a ~1.2-fold change (Fig. 6c, d). Similar results were observed irrespective of the absolute value of the open-loop controller’s output (Supplementary Fig. 26), and at a different initial concentration of the input *P*_*X*_ (Supplementary Fig. 27). These results suggest that our closed-loop controller might lead to improved robustness of in vitro biomolecular processes with respect to changes in temperature.

## Discussion

In this work, we constructed a synthetic biomolecular integral controller circuit for effective and robust control of the protein production rate of an output gene. We demonstrated that in the closed-loop configuration, the output followed the input signal linearly over a wide range of conditions, even under step changes in input. By harnessing the natural interaction between the *σ*_28_ and the anti-*σ*_28_ factor a strong sequestration reaction is realized that allowed an effective computation of the error between the reference and the output signal. This error signal is then integrated mathematically. Because of this, and as predicted by theory, the closed-loop controller suppresses the disturbances’ effect on the output, which were introduced in the biochemical species as a local disturbance or in the reaction environment as a global disturbance. In contrast, the open-loop controller is unable to suppress the disturbances, as noticed by the large variations of the output signal. This illustrates the advantage of closed-loop architectures, where an error computation is employed.

Mathematical models play an important role in understanding complex synthetic networks. They provide insight into the operation of networks, and serve to guide experimentation. Here, we developed an ODE model for the integral controller that quantitatively explains the transient as well as the steady-state behavior of all the species involved in the system. We were able to obtain an effective model parameterization, by isolating parameters for each set of experimental data before finding their optimum values to achieve the best fit. Even though the presented model is a coarse-grained mechanistic model, it enabled us to explain the measured response of the controller, and can also predict dynamic trajectories for a wide range of operating conditions accurately. Based on the extracted parameter values, we derived a simplified version of the original model that is as effective as the original model and can be used for further theoretical analysis in order to gain deeper insight into the operation of the controller.

In this work, we assumed that the TXTL reactions have an unlimited source of energy for the first 8 h in the range of plasmid concentrations used. This allowed us to ignore the depletion of energy resources while building the ODE model of the controller. After 8 h, we observed a reduction in deGFP expression, suggesting a decrease of transcription and translation rates consistent with the depletion of energy resources and biochemical changes such as pH drop^25^. To account for the resource competition and depletion in the measured response, we were able to modify the ODE model in a such a manner that it was able to follow the experimental data beyond 8 h (see Supplementary Note 2). Future work could incorporate further validation and extension of this model. For example, any cell-free reaction kit has a lifetime as it accumulates byproducts and consumes the nutrients (ribonucleosides and amino acids) even in the absence of any DNA^41^. Because of this reason, we had to add the DNA before 6 h after the beginning of the reaction. Moreover, because the controller was implemented in a TXTL reaction platform at the scale of a few microliters, a deterministic model was used while ignoring the biological noise. For in vivo applications, it may be desirable to extend the deterministic model to a stochastic model to consider intrinsic biological noise^38^.

We used a molecular sequestration reaction to implement the error computation. Previous models used similar approaches to realize closed-loop controllers for reference tracking and disturbance rejection^13–18^. In particular, recent experimental work demonstrated new capabilities of the closed-loop controller^17–19^. Our design, while related to those in^13,17,18,27^, has advantageous features which allowed us to carry out experimentation, much beyond what was previously possible, with variations in parameters and conditions. In addition, our results are unique in the sense that we have a quantitatively well-characterized controller that is constructed in all *E. coli* TXTL system, where biological noise is negligible compared to in vivo implementations. In the latter, biological noise plays a non-negligible role in governing the system dynamics^18^. Because of this, we cannot only precisely regulate gene expression rate, but can also accurately predict the dynamic response of the integral controller. Our controller design, which uses a gene network, can in principle also be applied to regulate any other genes of interest, such as those involved in controlling metabolic rates, a task which might not be feasible using a post-transcriptional based controller^13^. We also demonstrate disturbance rejection capabilities of our controller when a constant or a step disturbance is added to DNA concentration ($P_Y^{{\mathrm{tot}}}$ and $P_Z^{{\mathrm{tot}}}$); disturbances in DNA concentration are realistic because they typically arise in in vivo or in vitro genetic networks. Such experiments are not feasible using an in vivo reaction platform, thereby limiting the usage of the controller in rapid prototyping and implementation.

Notably, for the current architecture, disturbance rejection is only possible when perturbations do not directly influence the parameters involved in governing the production rate of the reporter protein and the protein *X* is expressed at values that are below or of order *ν/ω* (shown in Fig. 4d). These parameters are the concentration of *y* and *z* genes, and the association (*ω*) and dissociation (*ν*) rates. We have shown the controller capability to minimize the effect of the disturbance added on the concentration of $P_Y^{{\mathrm{tot}}}$ and *P*_*Z*_^tot^. To demonstrate robustness with respect to the transcriptional activation rates, we have conducted *in-silico* experiments in which we modified *ω* and *ν* parameters from their nominal values and observed a small variation in the closed-loop output compared to a significant variation in the open-loop output (Supplementary Figs. 30 and 31).

While adding disturbances in the biochemical species or in the reaction parameters, we observed that the steady-state output value was perturbed by a non-negligible amount (>10%) at 8 h. This may be due to the limited effectiveness of the error computation. In our design, effective error computation requires that *X* sequesters *Y* rapidly compared to the other reactions which use *X*, namely the two transcriptional activation reactions. If the consumption of *X* is not dominated by the sequestration reaction compared to the retroactivity effect^42^ due to the transcriptional activation reactions, the controller’s performance is degraded, and the output has a limited capacity to track the reference signal accurately on the face of disturbances. We, therefore, hypothesized that in the current controller, the effect of the disturbance on the output can be reduced but not completely rejected because of the slight ineffectiveness of error computation, which is due to the limited sequestration reaction rate constant. To test this, we have conducted *in-silico* experiments in which we increased the molecular sequestration rate while keeping the nominal transcriptional activation rate fixed and found that the variation in the steady-state output was reduced from 10% to <0.5% at 12 h (Supplementary Fig. 32a). Alternatively, when we increased the transcription activation rate while keeping the nominal sequestration rate fixed, the steady-state output was increased from 10% to ~30% as a function of *P*_*Y*_^tot^ and *P*_*Z*_^tot^ (Supplementary Fig. 32b).

It is important to note that our controller uses the expression of gene *z* as a read-out signal. Therefore, in order to ensure that the plant signal is recorded accurately through *Z*, the concentration of gene *z* must be the same as that of gene *y* $(P_Y^{{\mathrm{tot}}} = P_Z^{{\mathrm{tot}}})$ and the same promoters must be used on *y* and *z* genes so that the association and dissociation rates for the transactional activator are the same. These conditions ensure that at any time during the reaction, $P_Z^ + = P_Y^ +$. To achieve this operating condition in vivo, *Y* and *Z* could be expressed on the same operon, controlled by a single promoter.

Finally, in this work, we demonstrated that the closed-loop controller can robustly control the single gene expression rate of the deGFP fluorescent reporter protein taken as a model process to be controlled. However, we anticipate that the controller design could be extended to tightly regulate multiple genes that encode other biologically relevant proteins simultaneously or could be employed within a complex network system where multiple processes required tight regulation to improve robustness and performance of the network.

Molecular controllers capable of robust gene regulation are needed in synthetic biology in order to implement more complex circuit networks. The well-characterized and rationally implemented synthetic integral feedback controller we presented here is capable of addressing these challenges to advance biological engineering, and could lead to the development of powerful, synthetic network systems capable of achieving complexity similar to that found at the cellular level, to develop cell-free applications such as calibrated biomanufacturing or programming synthetic cells for specific tasks.

## Methods

### Mathematical modeling and parameter estimation

The simulated response of the controller was determined by numerically integrating ODE models (Fig. 3b) using the MATLAB ode23s solver unless otherwise specified. Initial conditions for each molecular species are described in the figure captions, and the values of reaction parameters are shown in Table 1. For the cases where there is a step change in the DNA concentration over the course of the reaction, similar settings were used to determine the model response numerically.

For parameter estimation, first we found initial guesses of parameter values that qualitatively agreed with the measured open-loop response. We then randomly sampled a set of input parameters from a uniform distribution within a bounded interval (upper and lower bounds of 15% each) centered around the initial guess values. This input set of parameters was then optimized to minimize the error between the model and measured open-loop responses for all five trajectories (shown in Fig. 3c). To find the best fit, the least squares error between the model and the measured response was minimized using the MATLAB fmincon function. During the fitting, each input parameter was allowed to vary from 0.1 to 10 times with respect to the input value. Further constraints were placed on the parameters so that they lie within a feasible biological range. For example, the activated transcriptional rate must be several orders of magnitude larger than the basal expression (*α*_*V*_^*+*^»*α*_*V*_ and *α*_*W*_^*+*^»*α*_*W*_) and the transcriptional rates of the *x* gene and activated *y* and *z* genes should be in the same order (*α*_*U*_ ≈ *α*_*V*_^*+*^ ≈ *α*_*W*_^*+*^) (see Supplementary Table 1). Because in the open-loop case, a modified version of *y* gene (denoted as *yc*) is expressed from the promoter $P_{YC}^{{\mathrm{tot}}}$ and cannot sequester with *X*, parameters *α*_*V*_, *α*_*V*_^*+*^, *δ*_*V*,_ *β*_*Y*_, *κ*, *κ*_i_, and $P_Y^{{\mathrm{tot}}}$ were set to zero. Once we found the optimum set of parameter values that provided the best fit for the open-loop response, these parameter values were then fixed during fitting all five trajectories of the closed-loop response (shown in Fig. 3d) while varying only *α*_*V*,_ *α*_*V*_^+^, *δ*_*V*,_ *β*_*Y*_, *κ*, and *κ*_i_. This resulted in a set of 16 parameters that fit both open and closed-loop responses (Fig. 3c, d, respectively). The fitting process was repeated 1000 times, which gave a range for the 16 parameters (Supplementary Fig. 7) with 95% confidence interval.

### TXTL reactions

The cell-free expression system used in this work is myTXTL from Arbor Biosciences. TXTL reactions were assembled using the Labcyte Echo 550 liquid handler, to volumes of 2 µl in a 96-well V-bottom plate (Corning Costar 3357 with caps Costar 3080) and incubated at 29 °C.

### DNA

Plasmids were constructed using standard cloning techniques. P_70a_ and P_28a_ are the strongest *E. coli σ*_70_ and *σ*_28_ promoters reported in TXTL, respectively. Each plasmid contains the untranslated region and RBS named UTR1, originally from the promoter 14 of the T7 phage genome, and either the *σ*_70_ promoter P_70a_ or the *σ*_28_ promoter P_28a_. UTR1 uses the strongest reported RBS in TXTL. All DNA constructs with terminators use the strong synthetic terminator T500. All the sequences of the plasmids and DNA parts (promoters and transcription terminators) are reported in the DNA sequences Supplementary File^24,25,36^. The *mSA* gene is 348 bp long, while *flgM* gene is 294 bp long. mSA is a soluble protein, like FlgM. The mSA protein does not have a regulatory effect on gene expression in these reactions and is used as a control for FlgM. For experiments with step changes in the concentration of DNA, the TXTL reactions were assembled in the same manner, using the Labcyte Echo 550, and incubated in a plate reader at 29 °C. Reactions were then taken out of the plate reader, and the additional DNA was added to the reaction using the Labcyte Echo 550. The well plate was then immediately returned to the plate reader. The total time that the well plate was out of the reader and at room temperature was less than two minutes. The step-change of DNA added to the reactions diluted the TXTL reaction by <5%.

### TXTL time-course fluorescence measurements

Fluorescence kinetics were performed using the reporter protein deGFP, a truncated version of eGFP that is more translatable in the TXTL system (25.4 kDa, 1 mg/mL = 39.38 µM)^24^. Measurements were carried out on Synergy H1 and Neo2 (Biotek Instruments) plate readers, using an excitation of 485 nm and emission of 525 nm, measuring every 3 min. To quantify the concentration of deGFP on the plate readers, a standard curve of intensity vs. deGFP concentration was made using recombinant eGFP (Cell Biolabs)^36^. All reactions were performed in at least triplicate.

### Reporting summary

Further information on research design is available in the Nature Research Reporting Summary linked to this article.

## Supplementary information

Supplementary Information

Reporting Summary

## Data Availability

The programming code that was used to analyze the raw data that supports the findings of this study are available from the corresponding author upon request.

## Source data

Source Data

## Data availability

The source data underlying Figs. 2–6, Supplementary Figs. 1–6, 8–10, 12, 15, 21–23, 25–27, 29 are provided as a Source Data file. All other data are available from the authors upon reasonable request.
