# Supplementary material for: In vitro implementation of robust gene regulation in a synthetic biomolecular integral controller
Source: Nat Commun. 2019 Dec 17;10:5760. doi: 10.1038/s41467-019-13626-z (PMC6917713; doi:10.1038/s41467-019-13626-z)
Supplement: Supplementary file 1 — Supplementary Information [file 41467_2019_13626_MOESM1_ESM.pdf]

Supplementary Information

In vitro implementation of robust gene regulation in a synthetic biomolecular integral controller

Agrawal *et al.*

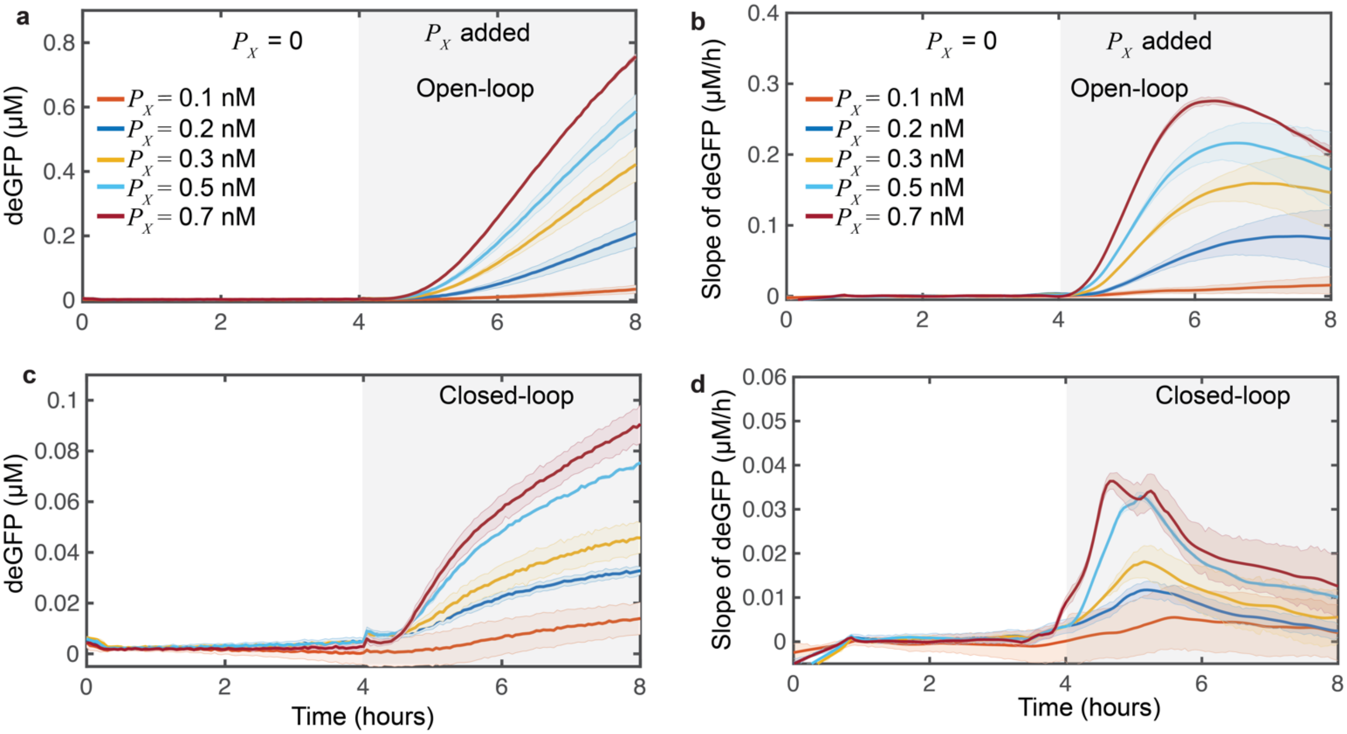

Supplementary Figure 1. Controller response for a step change in the input P_X. (a,c) Measured response for a step change in P_X, which was increased from 0 nM to different concentrations (0.1-0.7 nM) after 4 hours of the reaction in the presence of an initial 1 nM of P_Y^{tot} and P_Z^{tot} each. The corresponding deGFP slopes for the (b) open-loop and (d) closed-loop cases. To disable the feedback in the open-loop case P_Y^{tot} was replaced by P_{YC}^{tot}, which expresses a protein that cannot sequester with X (see Methods). Error bars are shown in the shaded region and were determined using the standard error of the mean of three or more repeats. A calibration factor was used to convert the measured deGFP fluorescent signal into the concentration. Before calculating deGFP slopes, measured deGFP responses were smoothed-out using the *rloess* smoothing method in MATLAB. Source data are provided as a Source Data file.

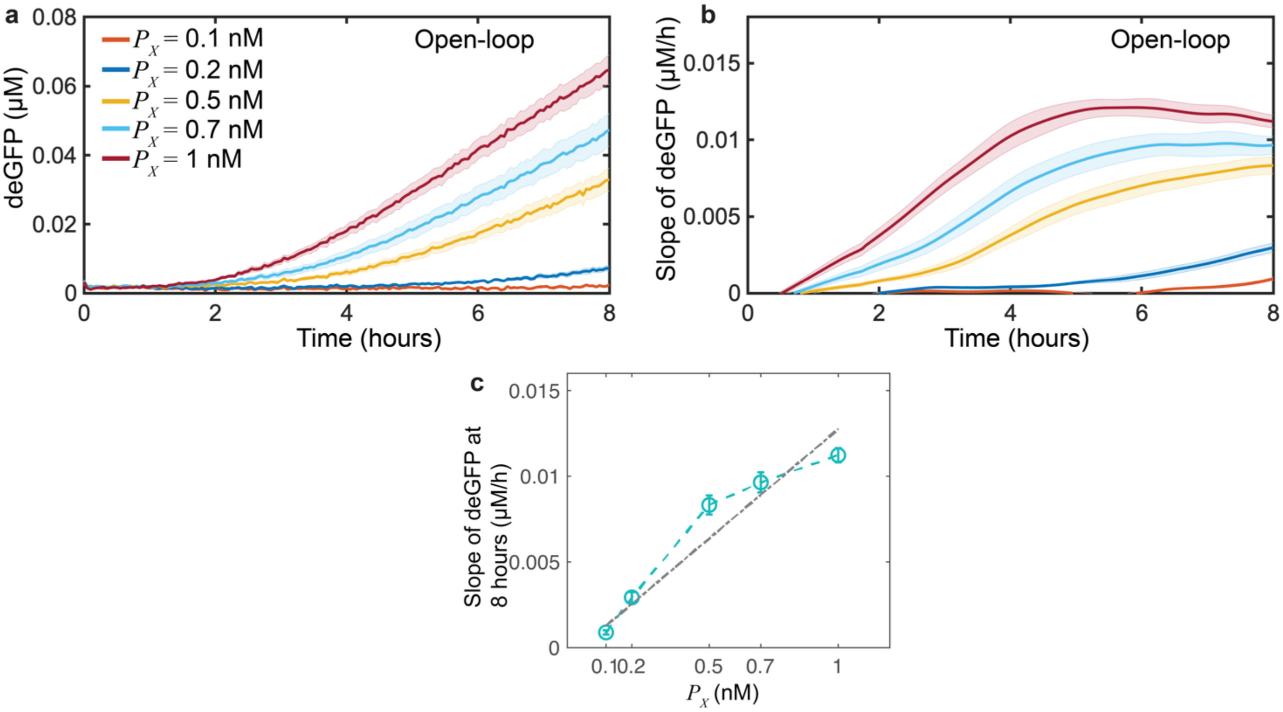

Supplementary Figure 2. Open-loop controller unable to track the reference signal independent of the absolute value of the output. Measured (a) deGFP response and (b) the corresponding slopes in the open-loop configuration at different initial concentrations of P_X (0.1 - 1 nM) while initial P_{YC}^{tot} and P_Z^{tot} were both 0.05 nM. (c) Summary of the deGFP slopes of the controller at 8 hours for a step change in P_X. A linear regression with zero intercept was used to fit the deGFP slopes and the corresponding R-square values is 0.89. Error bars are from the SEM of at least three repeats. A calibration factor was used to convert the measured deGFP fluorescent signal into the concentration. Before calculating deGFP slopes, measured deGFP responses were smoothed-out using the *rloess* smoothing method in MATLAB. Source data are provided as a Source Data file.

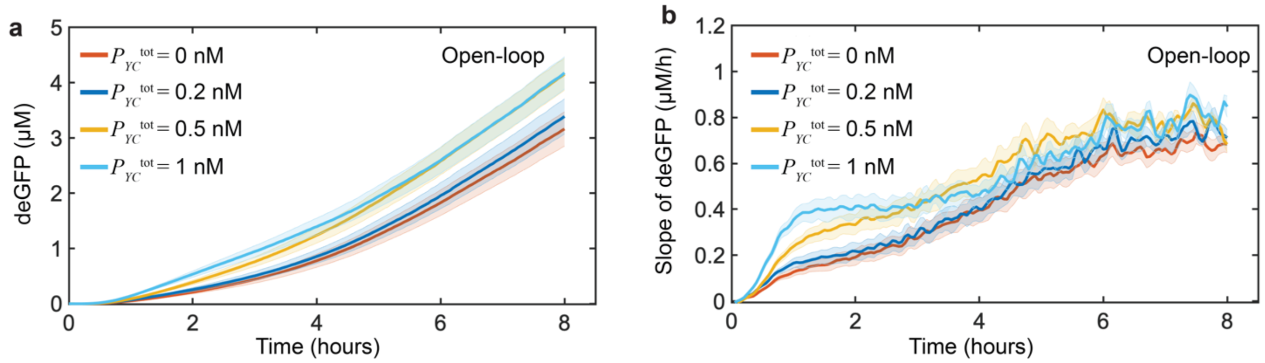

Supplementary Figure 3. Control gene *yc* does not affect the open-loop controller response. Measured (a) deGFP response and (b) the corresponding slope of the integral controller in the open-loop configuration at different initial concentrations of P_{YC}^{tot} (0 - 1 nM) while initial P_X and P_Z^{tot} were both 1 nM each. Error bars are from the standard error of the mean (SEM) of at least three repeats. Source data are provided as a Source Data file.

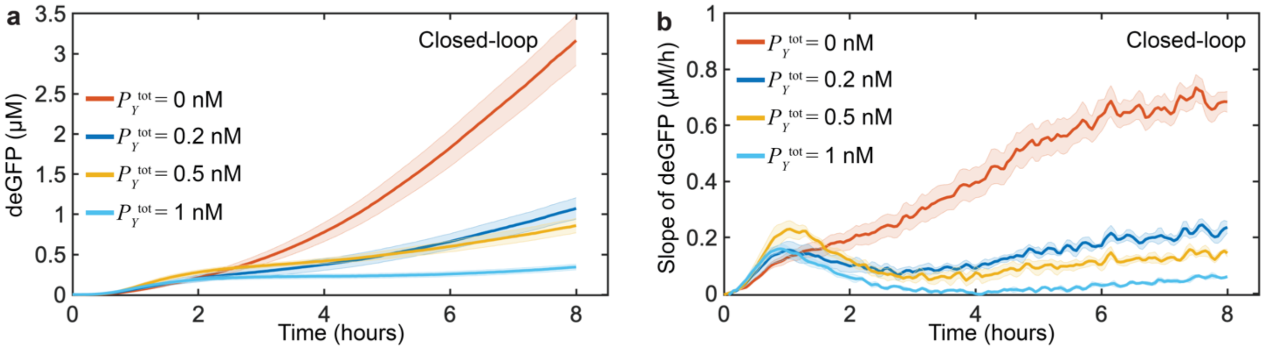

Supplementary Figure 4. An increase in gene *y* concentration leads to a reduction in the output. Measured (a) deGFP response and (b) the corresponding slope of the integral controller in the closed-loop configuration at different initial concentrations of P_Y^{tot} (0 - 1 nM) while initial P_X and P_Z^{tot} were both 1 nM each. Error bars are from the standard error of the mean (SEM) of at least three repeats. Source data are provided as a Source Data file.

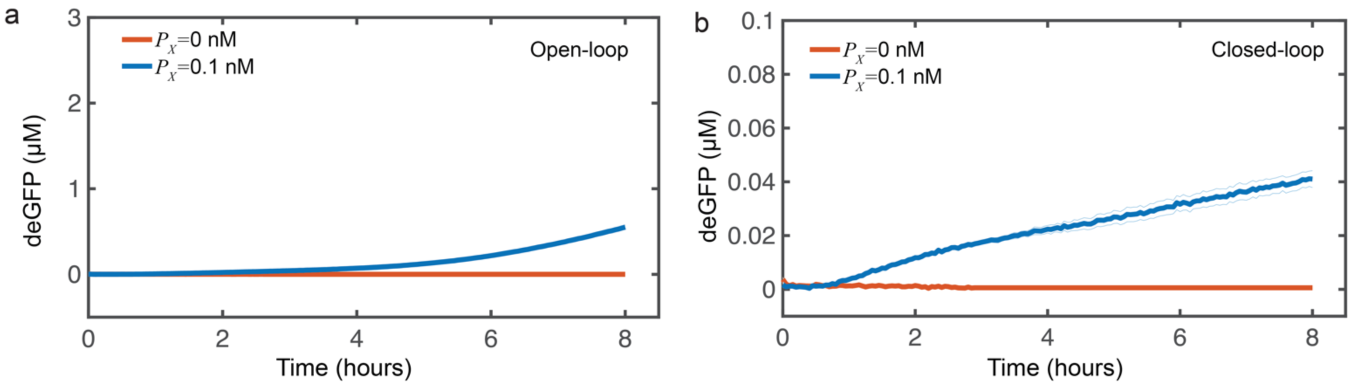

Supplementary Figure 5. In the absence of input P_X the controller response is negligible. Measured deGFP response of the integral controller in the (a) open-loop and (b) closed-loop configurations at two different initial concentrations of P_X (0 and 0.1 nM) while initial P_Y^{tot} and P_Z^{tot} were both 1 nM each. To disable the feedback in the open-loop case, P_Y^{tot} was replaced by P_{YC}^{tot}. Error bars are from the SEM of at least three repeats. Source data are provided as a Source Data file.

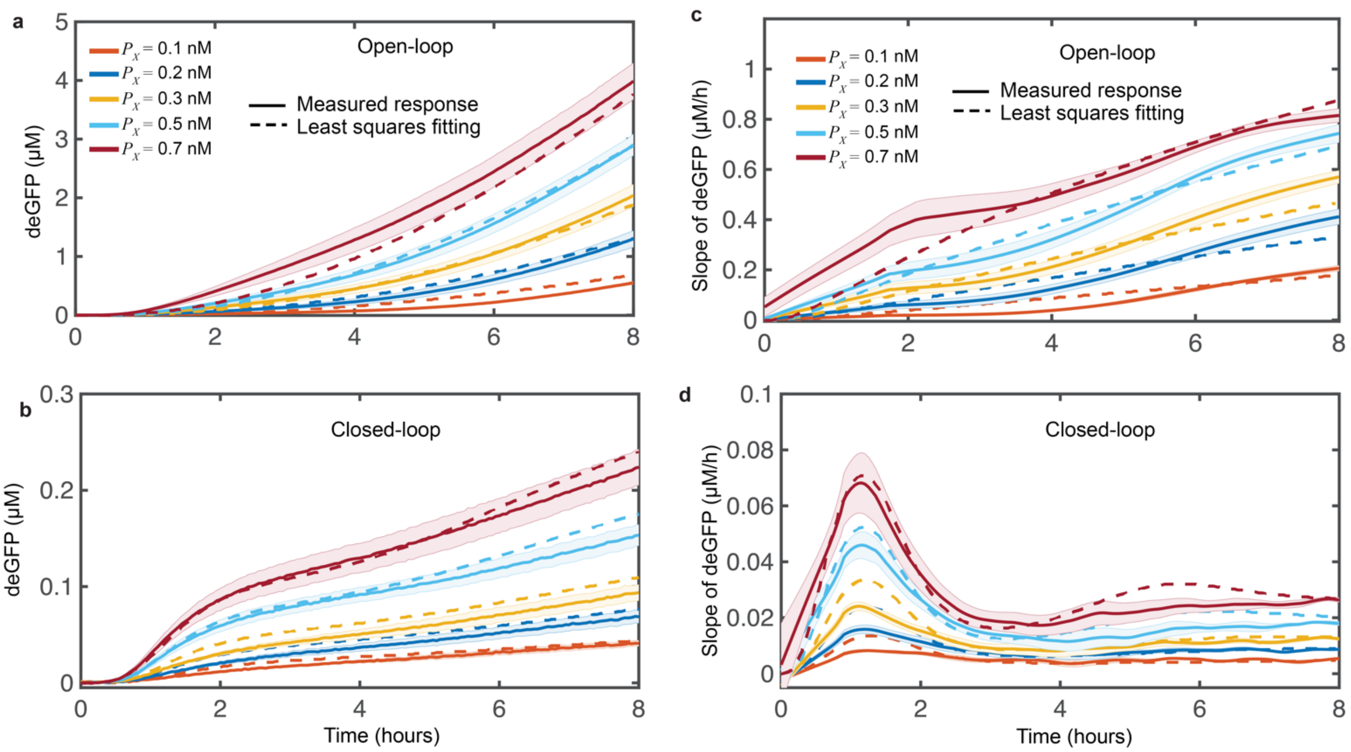

Supplementary Figure 6. **Results of the least squares fitting.** (a-b) Comparing the model response with the measured deGFP response of the integral controller in the (a) open-loop and (b) closed-loop configurations at different initial concentrations of P_X (0.1 - 0.7 nM) while initial P_Y^{tot} and P_Z^{tot} were both 1 nM. (c-d) Corresponding deGFP slopes for the (c) open-loop and (d) closed-loop operations. To disable the feedback in the open-loop case, P_Y^{tot} was replaced by P_{YC}^{tot}. Error bars are from the SEM of at least three repeats. The ODE model shown in Fig. 3b was used to determine the response with parameters shown in Table 1. Before calculating deGFP slopes, measured deGFP responses were smoothed-out using the *rloess* smoothing method in MATLAB. Source data are provided as a Source Data file.

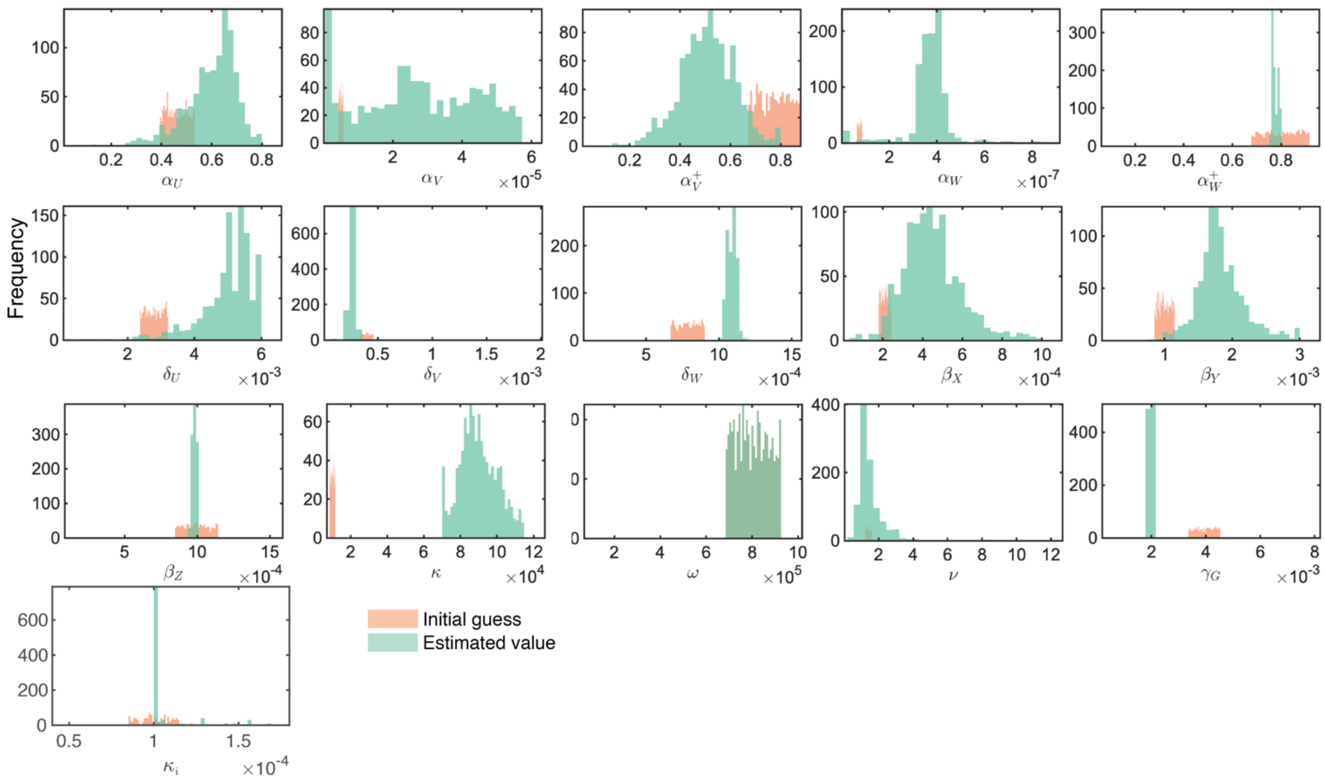

Supplementary Figure 7. Histograms of the initial guesses and estimated parameters were obtained from 1000 samples that gave the lowest fitting error (see Methods).

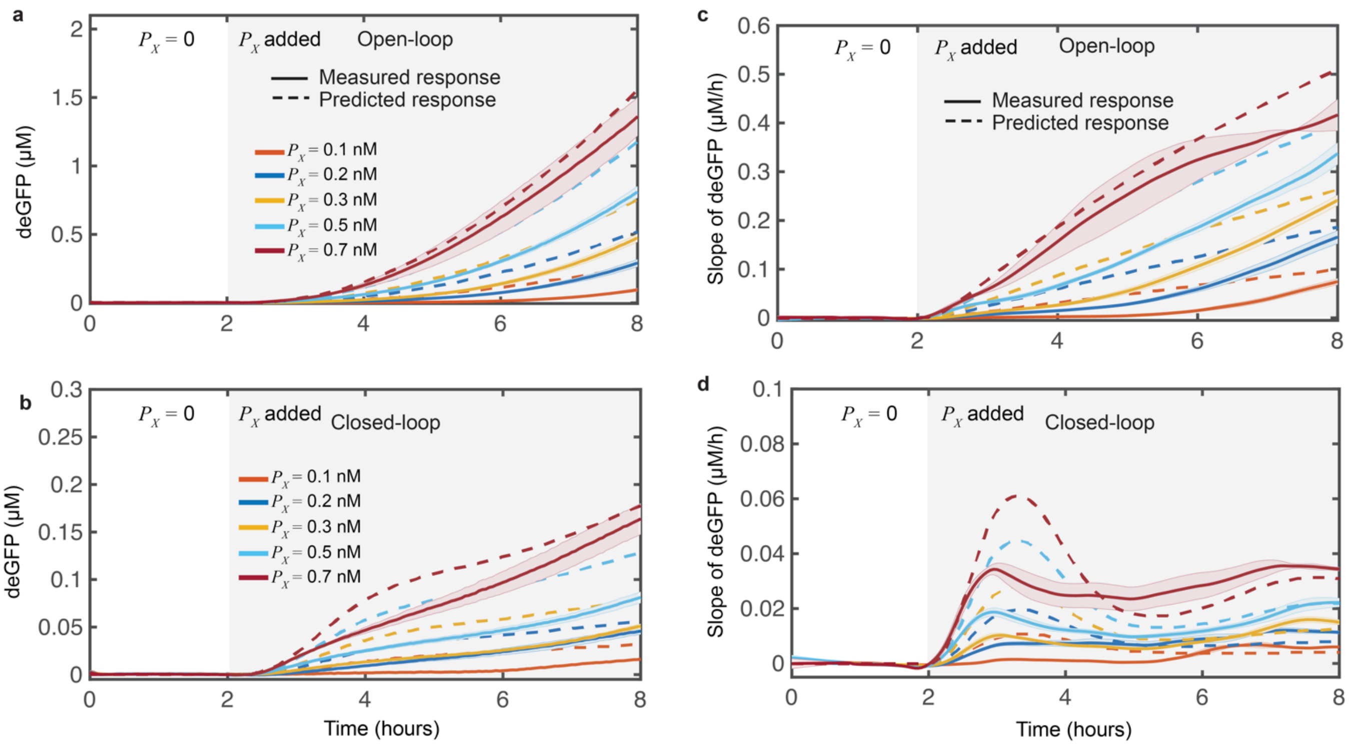

Supplementary Figure 8. Comparing the predicted response of the integral controller with the measured response in the (a) open-loop and (b) closed-loop configurations for a step change in P_X, and the corresponding deGFP slopes in (c) and (d) respectively. P_X was increased from 0 nM to different concentrations (0.1-0.7 nM) after 2 hours of the reaction in the presence of initial 0.7 nM of P_Y^{tot} and P_Z^{tot} each. To disable the feedback in the open-loop case, P_Y^{tot} was replaced by P_{YC}^{tot}. Error bars are from the SEM of at least three repeats. The ODE model shown in Fig. 3b was used to determine the response with parameters shown in Table 1. Before calculating deGFP slopes, measured deGFP responses were smoothed-out using the *rloess* smoothing method in MATLAB. Source data are provided as a Source Data file.

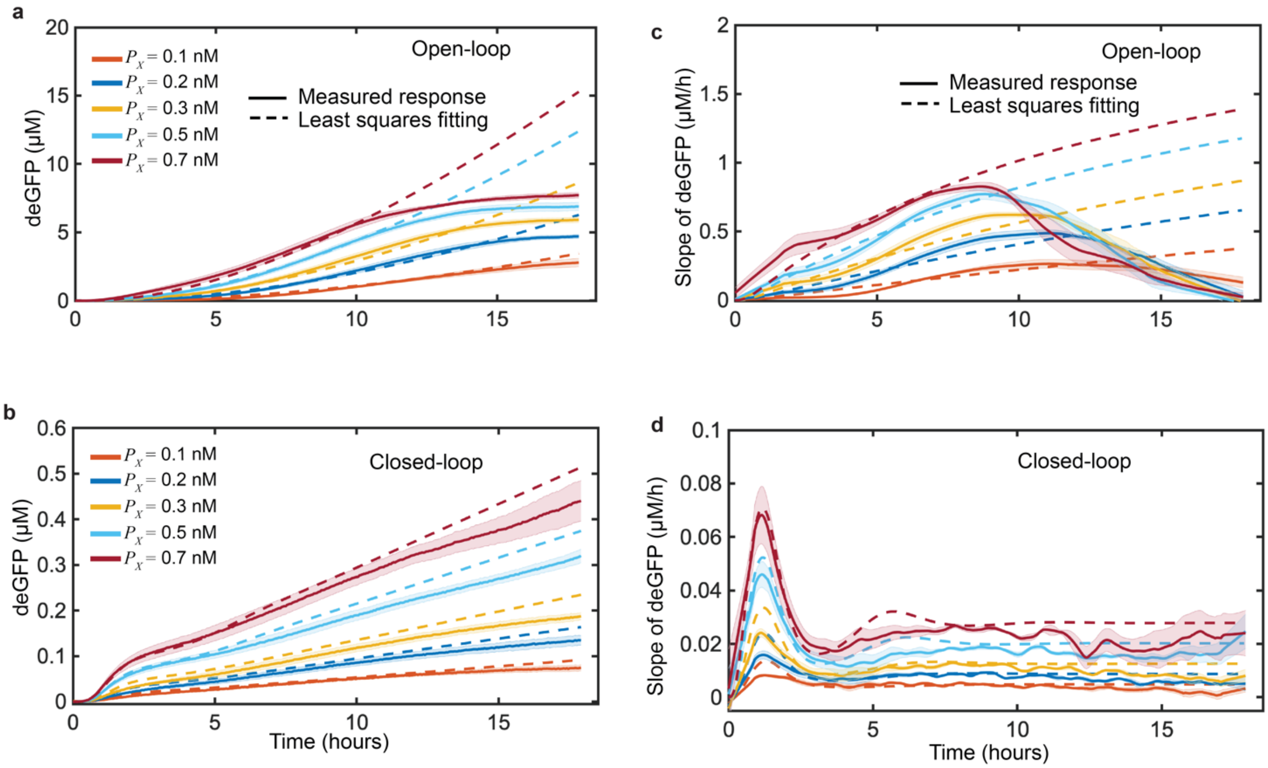

Supplementary Figure 9. **Effect of the resource limitation in the TXTL reaction.** (a-b) Comparing the model response with the measured deGFP response of the integral controller in the (a) open-loop and (b) closed-loop configurations at different initial concentrations of P_X (0.1 - 0.7 nM) while initial P_Y^{tot} and P_Z^{tot} were both 1 nM. (c-d) Corresponding deGFP slopes for the (c) open-loop and (d) closed-loop operations. To disable the feedback in the open-loop case, P_Y^{tot} was replaced by P_{YC}^{tot}. Error bars are from the SEM of at least three repeats. The ODE model shown in Fig. 3b was used to determine the response with parameters shown in Table 1. Measured open loop response starts saturating after 8 hours of the reaction, suggesting resource limitation in the reaction. After 8 hours, the reaction runs out of energy and is also limited by the degradation products accrued. Before calculating deGFP slopes, measured deGFP responses were smoothed-out using the *rloess* smoothing method in MATLAB. Source data are provided as a Source Data file.

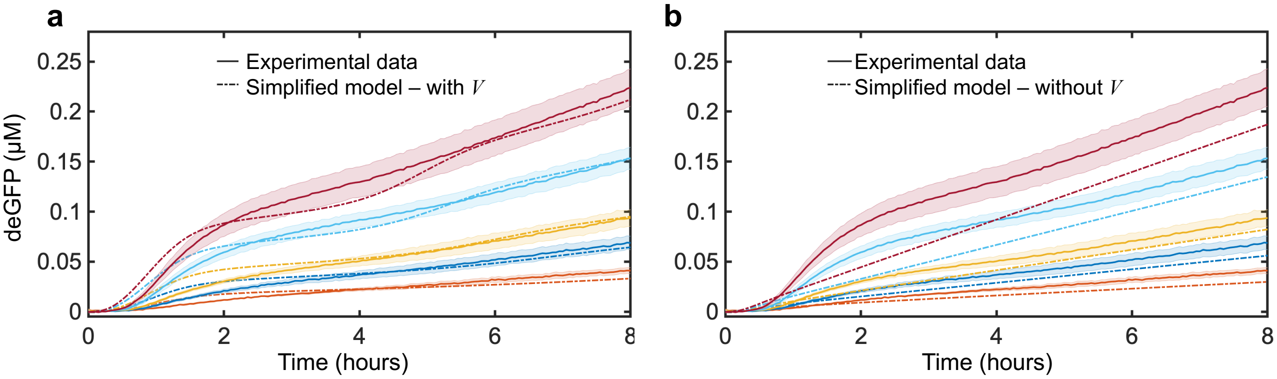

Supplementary Figure 10. Comparing the closed-loop simplified model response (a) with V and (b) without V at different initial concentrations of P_X (0.1 - 0.7 nM) while initial P_Y^{tot} and P_Z^{tot} were both 1 nM each. Error bars are from the SEM of at least three repeats. The ODE model shown in Fig. 4c was used to determine the model response with parameters shown in Table 1. In the simplified model without V, ODE of V was replaced by its steady-state value (see Note 1). Source data are provided as a Source Data file.

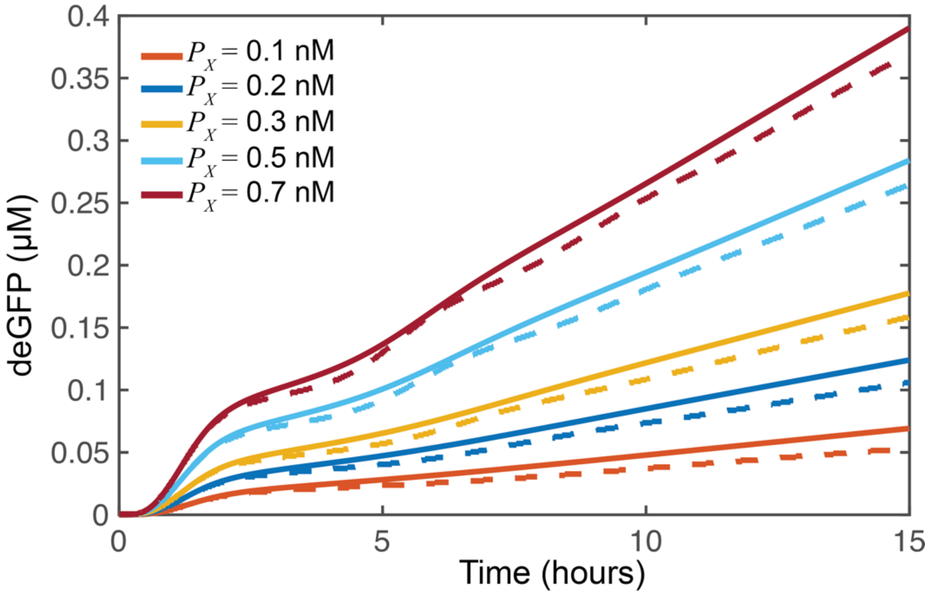

Supplementary Figure 11. Determining the effect of the reverse sequestration on the read-out signal at different initial concentrations of P_X (0.1 - 0.7 nM) while initial P_Y^{tot} and P_Z^{tot} were both 1 nM each. The ODE model shown in Fig. 3b was used to determine the model response with parameters shown in Table 1. Here the dashed-lines represent the cases where $\kappa_i = 0$.

Supplementary Note 1

From chemical reactions, we built an ODE model of the integral controller (see Fig. 3a):

$$\frac{dU}{dt} = \alpha_U P_X - \delta_U U, \tag{1}$$

$$\frac{dX}{dt} = \beta_X U - \kappa XY + \kappa_i [XY] - \omega X P_Y + \nu P_Y^+ - \omega X P_Z + \nu P_Z^+, \tag{2}$$

$$\frac{dP_Y^+}{dt} = \omega X P_Y - \nu P_Y^+, \tag{3}$$

$$\frac{dV}{dt} = \alpha_V P_Y + \alpha_V^+ P_Y^+ - \delta_V V, \tag{4}$$

$$\frac{dY}{dt} = \beta_Y V - \kappa XY + \kappa_i [XY], \tag{5}$$

$$\frac{d[XY]}{dt} = \kappa XY - \kappa_i [XY], \tag{6}$$

$$\frac{dW}{dt} = \alpha_W P_Z + \alpha_W^+ P_Z^+ - \delta_W W, \tag{7}$$

$$\frac{dP_Z^+}{dt} = \omega X P_Z - \nu P_Z^+, \tag{8}$$

$$\frac{dZ}{dt} = \beta_Z W - \gamma_G Z, \tag{9}$$

$$\frac{dG}{dt} = \gamma_G Z. \tag{10}$$

Here and elsewhere, subscripts to the parameters indicate the corresponding species. The parameters α and β are transcription and translation rates, respectively. Each mRNA species (U, V and W) has a degradation rate denoted as δ. The parameters κ and κ_i are the forward and reverse sequestration rates respectively, and the parameters ω and ν are the association and dissociation rates for the transcriptional activation reaction. The parameter γ is the maturation rate and α^+ represents increased transcription rate compared to α. Considering mass-conservation, we assume that $P_Y^{tot} = P_Y + P_Y^+$ and $P_Z^{tot} = P_Z + P_Z^+$ at all times.

We find experimentally and numerically that the transcriptional activation reactions (3) and (8) are much faster than the other reactions involved in the genetic network. Since both (3) and (8) are asymptotically stable, we used a quasi-steady-state approximation to replace the values of P_Y^+ and P_Z^+ by their quasi-steady-state approximations (see $\bar{P_C}$ below). Moreover, as the RNA dynamics is much faster than the protein dynamics (due to the faster RNA degradation rates), and (1), and (7) are asymptotically stable, a similar approximation was used to model the synthesis of X and Z using single reactions for each, while keeping the two-step synthesis of Y to ensure that we consider an appropriate delay in the overall system dynamics (Supplementary Fig. 10). We also ignore the reverse sequestration rate κ_i as it has a limited impact on the output dynamics (Supplementary Fig. 11). Ignoring (10), this leads to a simplified model for the controller:

$$\frac{dX}{dt} \approx \beta'_X P_X - \kappa XY, \tag{11}$$

$$\frac{dV}{dt} \approx \alpha_V^+ \bar{P_C} - \delta_V V, \tag{12}$$

$$\frac{dY}{dt} \approx \beta_Y V - \kappa XY, \tag{13}$$

$$\frac{dZ}{dt} \approx \beta'_Z \bar{P_C} - \gamma_G Z, \tag{14}$$

$$\bar{P_C} := \frac{X}{X + \frac{\nu}{\omega}} P_C^{tot}. \tag{15}$$

Here, $\beta'_X = \frac{\beta_X \alpha_U}{\delta_U}$, $\beta'_Z = \frac{\beta_Z \alpha_W^+}{\delta_W}$, $P_C^{tot} = P_Y^{tot} = P_Z^{tot}$, $P_C^{tot} = P_C + P_C^+$ and bar ($\bar{\ }$) denotes a quasi-steady-state value. We have validated this model by the comparing its response with the original model response, as shown in Supplementary Fig. 12. Note that as long as the protein X is expressed at values that are below or of order $\frac{\nu}{\omega}$, $\bar{P_C} < P_C^{tot}$, which means that the y and z genes have free promoter sites where X can bind to increase the production rates of Y and Z. If, instead, $X \gg \frac{\nu}{\omega}$, then $\bar{P_C}$ is approximately equal to P_C^{tot}, which means $\bar{P_C}$ is independent of X, and so are Y and Z.

Analytical solution for deGFP slopes for the open and closed-loop cases

To determine an analytical approximation to the steady-state for the closed-loop controller's output, the right-hand sides of (11) -(14) were set to zero:

$$0 = \beta'_X P_X - \kappa XY, \tag{16}$$
$$0 = \alpha_V^+ \bar{P_C} - \delta_V V, \tag{17}$$
$$0 = \beta_Y V - \kappa XY, \tag{18}$$
$$0 = \beta'_Z \bar{P_C} - \gamma_G Z. \tag{19}$$

Comparing (16) and (18) leads to:

$$\bar{V} \approx \frac{\beta'_X}{\beta_Y} P_X. \tag{20}$$

Using (17), (19), and (20), it can be shown that:

$$\bar{Z} \approx \frac{\beta'_Z}{\gamma_G} \frac{\beta'_X}{\beta_Y} \frac{\delta_V}{\alpha_V^+} P_X. \tag{21}$$

Using (10) and (21), it can be shown that:

$$\frac{dG}{dt} \approx \beta'_Z \frac{\beta'_X}{\beta_Y} \frac{\delta_V}{\alpha_V^+} P_X. \tag{22}$$

It should be noted that (22) is valid as long as enough promoter sites are available on the y and z genes, in other words, if the protein X is expressed at values that are below or of order $\frac{\nu}{\omega}$. For the cases where $X \gg \frac{\nu}{\omega}$ is true, $\bar{P_C} \approx P_C^{tot}$, and this leads to:

$$\bar{Z} \approx \frac{\beta'_Z}{\gamma_G} P_C^{tot}. \tag{23}$$

and therefore,

$$\frac{dG}{dt} \approx \beta'_Z P_C^{tot}. \tag{24}$$

Note that (24) means that the reference tracking is not achieved, and also the output becomes heavily dependent on perturbations. Thus, tracking and robustness are traded off depending on the range of $X(t)$. Now to determine the approximate analytical solution for the open-loop controller's output, we can ignore (12) and (13) as the y gene is absent in the open-loop operation, and so V and Y are not expressed. This leads to:

$$\frac{dX}{dt} \approx \beta'_X P_X, \tag{25}$$
$$\frac{dZ}{dt} \approx \beta'_Z \bar{P_C} - \gamma_G Z, \tag{26}$$
$$\bar{P_C} \approx \frac{X}{X + \frac{\nu}{\omega}} P_C^{tot}. \tag{27}$$

Assuming at time $t = 0$, $X = X_0$, and (25) can be written as:

$$X(t) \approx \beta'_X P_X t + X_0. \tag{28}$$

At the steady-state,

$$\bar{Z} \approx \frac{\beta'_Z}{\gamma_G} \bar{P_C}. \tag{29}$$

Using (27) -(29), and (10), it can be shown that:

$$\frac{dG}{dt} \approx \beta'_Z \frac{\beta'_X(\beta'_X P_X t + X_0)}{\beta'_X(\beta'_X P_X t + X_0) + \frac{\nu}{\omega}} P_C^{tot}. \tag{30}$$

From this analysis, it can be inferred that only the closed-loop controller's output can follow the reference signal linearly, which is the scaled value of P_X (22), as long as the protein X is expressed at values that are below or of order $\frac{\nu}{\omega}$. In contrast, in the open-loop case, a nonlinear dependence of the output on X can be seen (30). Moreover, in our implementation, Z is directly related the reported protein deGFP and because of that, in the analytical analysis, we used time derivative of G (scaled value of Z (10)) as a comparison metric instead of the plant signal (V), which is not a measurable quantity.

Analytical solution for plant signal V

The simplified model of the controller:

$$\frac{dX}{dt} \approx \beta'_X P_X - \kappa XY, \tag{31}$$

$$\frac{dV}{dt} \approx \alpha_V^+ \bar{P}_Y^+ - \delta_V V, \tag{32}$$

$$\frac{dY}{dt} \approx \beta_Y V - \kappa XY, \tag{33}$$

$$\frac{dZ}{dt} \approx \beta'_Z \bar{P}_Z^+ - \gamma_G Z, \tag{34}$$

$$\bar{P}_{Y^+} := \frac{X}{X + \frac{\nu}{\omega}} P_Y^{tot}, \tag{35}$$

$$\bar{P}_{Z^+} := \frac{X}{X + \frac{\nu}{\omega}} P_Z^{tot}. \tag{36}$$

Similar to the analysis above, when the closed-loop controller is at the steady-state, it can be shown that:

$$\bar{V} \approx \frac{\alpha_V^+}{\delta_V} \bar{P}_Y^+ \approx \frac{\beta'_X}{\beta_Y} P_X. \tag{37}$$

Note that the steady-state value of V ($\bar{V}$) is linearly proportional to the input P_X and independent of the concentration of y genes (P_Y^{tot}), and the association (ω) and dissociation (ν) rates. Thereby, the plant signal tracks the input P_X robustly. This is true when the protein X is expressed at values that are below or of order $\frac{\nu}{\omega}$, so that $\bar{P}_Y^+ < P_Y^{tot}$. Using (37), it can be shown that:

$$\bar{P}_Y^+ \approx \frac{\beta'_X}{\beta_Y} \frac{\delta_V}{\alpha_V^+} P_X. \tag{38}$$

From (35) and (36), it can be inferred that when $P_Z^{tot} = P_Y^{tot}$, $\bar{P}_Z^+ = \bar{P}_Y^+$. Steady-state value of Z is

$$\bar{Z} \approx \frac{\beta'_Z}{\gamma_G} \bar{P}_Z^+. \tag{39}$$

Now using (37) - (39), and the fact that $\bar{P}_Z^+ = \bar{P}_Y^+$, it can be shown that:

$$\bar{Z} \approx \frac{\beta'_Z}{\gamma_G} \frac{\delta_V}{\alpha_V^+} \bar{V} \approx \frac{\beta'_Z}{\gamma_G} \frac{\beta'_X}{\beta_Y} \frac{\delta_V}{\alpha_V^+} P_X. \tag{40}$$

Hence, the steady-state output of the controller $\bar{Z}$ is linearly proportional to the plant signal ($\bar{V}$) and so the input P_X. We can, therefore, use Z to read the plant signal.

Analytical equation for $X_\mathbf{R}$

Now to determine an analytical approximation of the error signal (X_R), which is $X - Y$, (13) was subtracted from (11):

$$\frac{d}{dt}(X - Y) \approx \beta'_X P_X - \beta_Y V(t). \tag{41}$$

This leads to:

$$X_\mathrm{R} = X - Y \approx \int_0^t (\beta'_X P_X - \beta_Y V(\tau)) d\tau. \tag{42}$$

Now using (20), it can be shown that:

$$X_\mathrm{R} = X - Y \approx \beta_Y \int_0^t \bar{V} - V(\tau) d\tau. \tag{43}$$

Therefore, the error signal is a mathematical integral of the difference between the steady-state ($\bar{V}$) and time dependent values (V) of the plant signal.

Local stability analysis

We can also evaluate the stability of the steady-state response of the closed-loop controller to local perturbations through local stability analysis. For simplicity, we ignore the reactions associated with z gene as they are not actively involved in the closed-loop dynamics (Fig. 1a and b). Moreover, assuming the protein X is expressed at values that are below or of order $\frac{\nu}{\omega}$ (for the linear reference tracking), the simplified model (11)-(15) can be reduced to:

$$\frac{dX}{dt} \approx \beta'_X P_X - \kappa XY, \tag{44}$$

$$\frac{dV}{dt} \approx \beta_1 X - \delta_V V, \tag{45}$$

$$\frac{dY}{dt} \approx \beta_Y V - \kappa XY. \tag{46}$$

where $\beta_1 = \alpha_V^+ P_C^{tot} \omega/\nu$. It can be shown that at the steady-state:

$$\bar{X} \approx \frac{\delta_V \beta'_X P_X}{\beta_Y \beta_1}, \tag{47}$$

$$\bar{V} \approx \frac{\beta'_X P_X}{\beta_Y}, \tag{48}$$

$$\bar{Y} \approx \frac{\beta_1 \beta_Y}{\delta_V \kappa}. \tag{49}$$

Now we can calculate the Jacobian for the model shown in (44)-(46) at the steady-state shown in (47)-(49):

$$\begin{bmatrix} \frac{-\beta_Y \beta_1}{\delta_V} & 0 & -\frac{\kappa \delta_V \beta'_X P_X}{\beta_Y \beta_1} \\ \beta_1 & -\delta_V & 0 \\ -\frac{\beta_Y \beta_1}{\delta_V} & \beta_Y & -\frac{\kappa \delta_V \beta'_X P_X}{\beta_Y \beta_1} \end{bmatrix}. \tag{50}$$

It can be shown that the characteristic equation of the Jacobian is:

$$s^3 + \left(\frac{\kappa \beta_X \bar{U} \delta_V}{\beta_1 \beta_Y} + \frac{\beta_1 \beta_Y}{\delta_V} + \delta_V\right) s^2 + \left(\frac{\delta_V^2 \kappa}{\beta_1 \beta_Y} + \beta_1 \beta_Y\right) s + \delta_V \kappa \beta_X P_X = 0. \tag{51}$$

Using the Routh-Hurwitz criterion[1], one can verify the stability of the system if the first column of the Routh array is positive. For (51), it can be shown that this is true when:

$$(B + \frac{\delta_V A}{B})(\frac{B}{\delta_V} + \frac{A}{B\delta_V}) - A > 0. \tag{52}$$

Here, $A = \delta_V \kappa \beta'_X P_X, B = \beta_1 \beta_Y$. Equation (52) is true for any values A, B and δ.

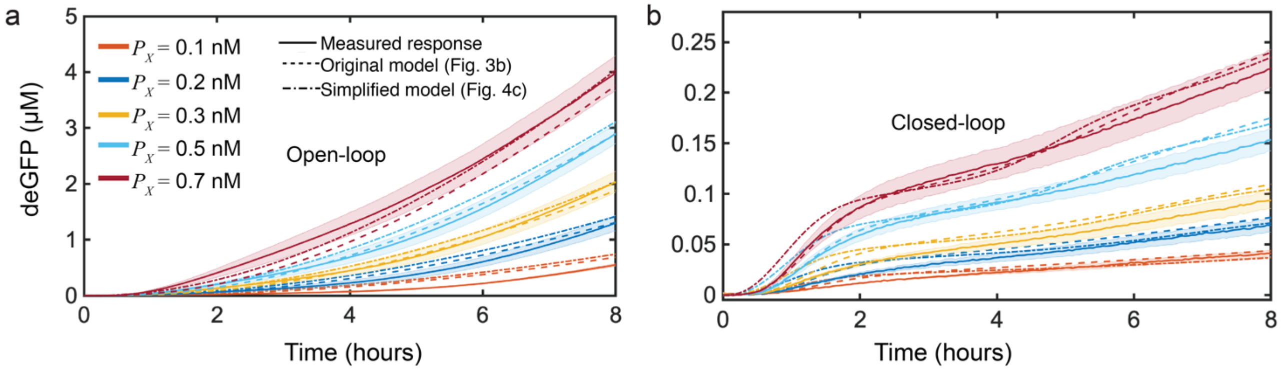

Supplementary Figure 12. Comparing the simplified model response with the original model in the (a) open-loop and (b) closed-loop configurations at different initial concentrations of P_X (0.1 - 0.7 nM) while initial P_Y^{tot} and P_Z^{tot} were both 1 nM each. To disable the feedback in the open-loop case, P_Y^{tot} was replaced by P_{YC}^{tot}. Error bars are from the SEM of at least three repeats. The ODE model shown in Fig. 3b was used to determine the original model response while the ODE model shown in Fig. 4c was used to determine the simplified model response with parameters shown in Table 1. Source data are provided as a Source Data file.

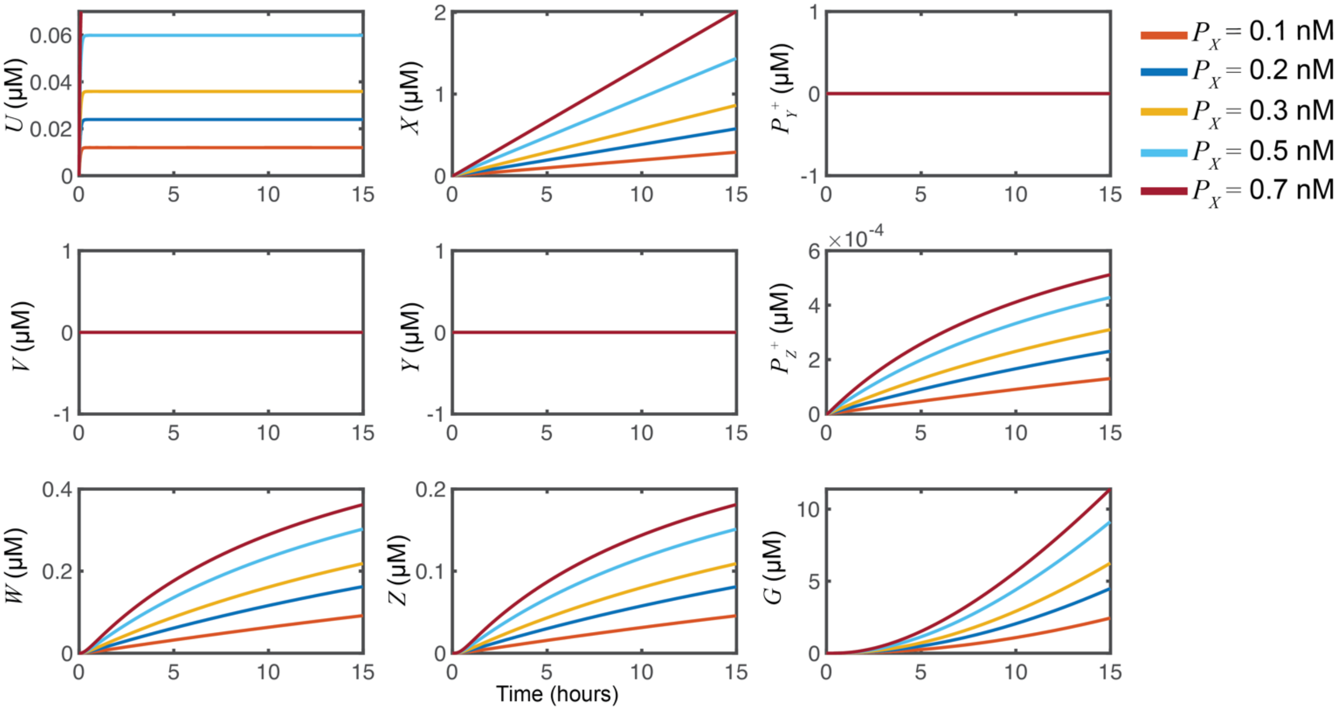

Supplementary Figure 13. Open-loop controller's output has no steady-state. Simulated open-loop response of the integral controller at different initial concentrations of P_X (0.1 - 0.7 nM) while initial P_Z^{tot} was 1 nM. The ODE model shown in Fig. 3b was used to determine the response with parameters shown in Table 1. The X protein does not consume over time in the open-loop case which causes Z to increase over time.

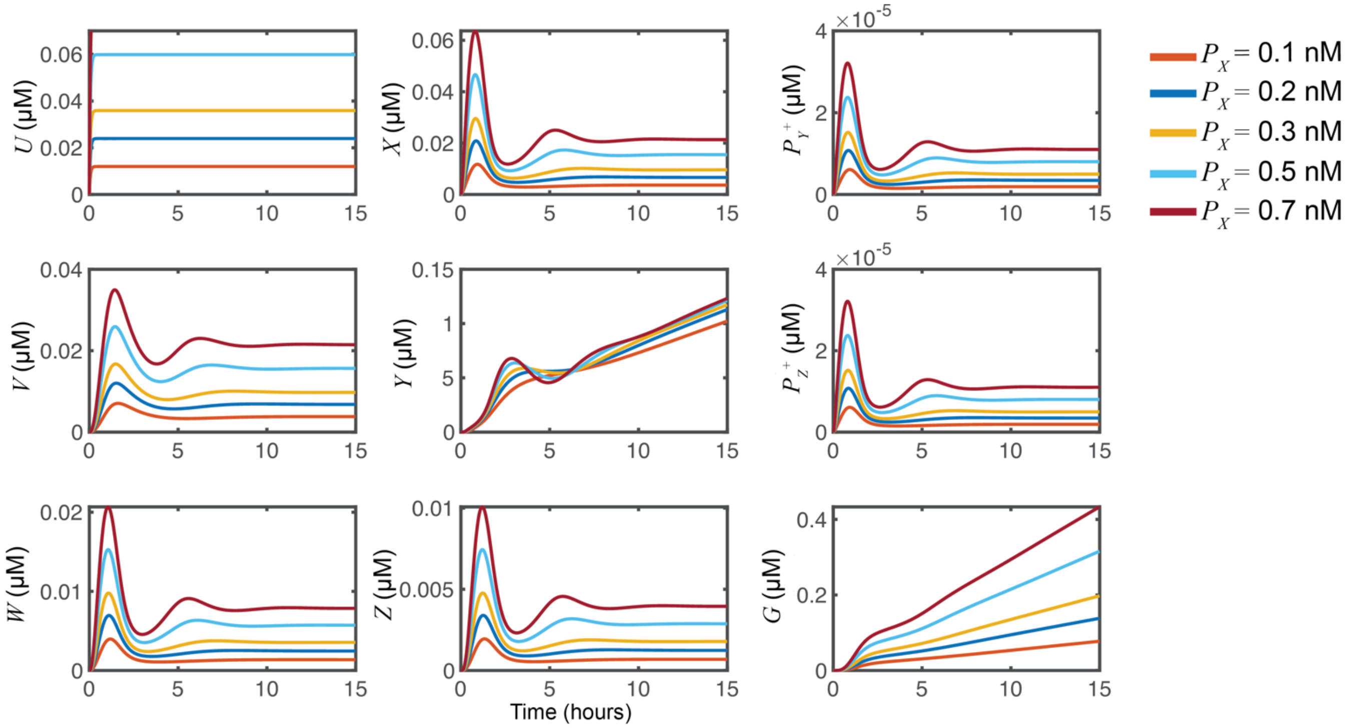

Supplementary Figure 14. Closed-loop controller's output has a steady-state. Simulated closed-loop response of the integral controller at different initial concentrations of P_X (0.1 - 0.7 nM) while initial P_Y^{tot} and P_Z^{tot} were both 1 nM each. The ODE model shown in Fig. 3b was used to determine the response with parameters shown in Table 1. Simulated response suggests that all the molecular species (except *Y* and *G*) involved in the genetic network approaches a steady-state at 8 hours. The slope of the measured response, which is the scaled value of *Z*, can be used to infer a steady-state behavior in the genetic network when operated in the closed-loop configuration.

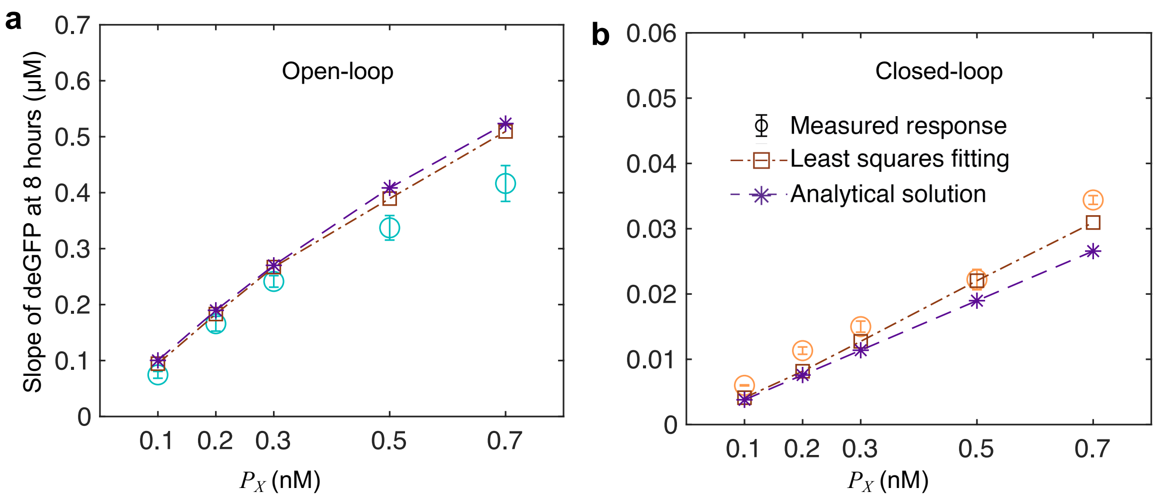

Supplementary Figure 15. Comparing the responses determined using the approximate analytical solution (Fig. 4d) with the original model response for the (a) open and (b) closed-loop cases for a step change in P_X, which was increased from 0 nM to different concentrations (0.1-0.7 nM) after 2 hours of the reaction in the presence of initial 0.7 nM of P_Y^{tot} and P_Z^{tot} each. To disable the feedback in the open-loop case, P_Y^{tot} was replaced by P_{YC}^{tot}. Error bars are from the SEM of at least three repeats. The ODE model shown in Fig. 3b was used to determine the response with parameters shown in Table 1. Before calculating deGFP slopes, measured deGFP responses were smoothed-out using the *rloess* smoothing method in MATLAB. Source data are provided as a Source Data file.

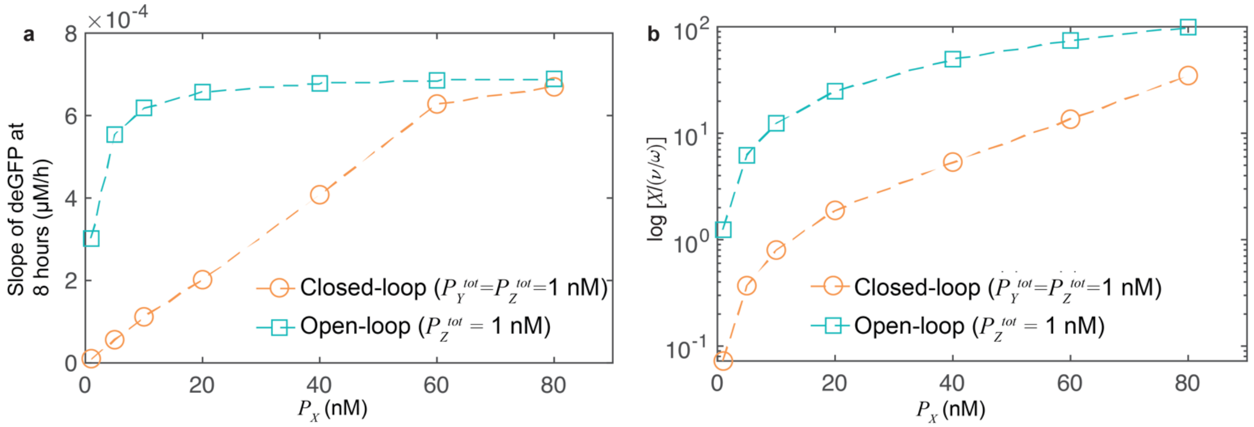

Supplementary Figure 16. Fundamental limits on parameters of the closed-loop controller for the output to track the input Px. Simulated controller response in the open-loop and closed-loop cases at different initial concentrations of P_X (1 - 80 nM). (a) Summary of the deGFP slopes at 8 hours and the corresponding values of (b) $X/(\nu/\omega)$. The ODE model shown in Fig. 3b was used to determine the response with parameters shown in Table 1. The simulations were conducted for 8 hours, and the maximum value of $X(t)$ was used to determine the value of $X/(\nu/\omega)$. deGFP slopes were calculated from Z using (10) mentioned in Note 1. The time derivative of G follows the input P_X linearly only for the closed-loop case as long as enough promoter sites are available on y and z genes, in other words (d) if the protein X is expressed at values that are below or of order ν/ω.

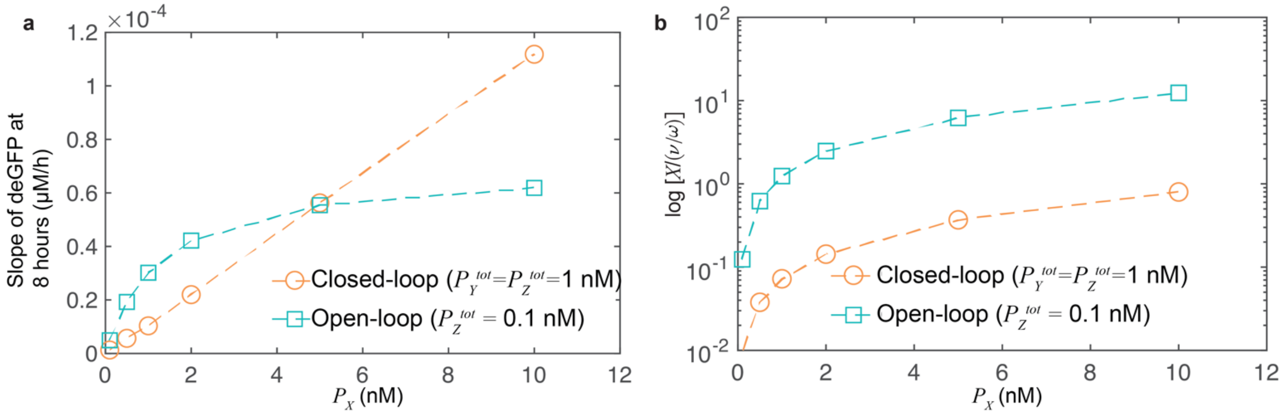

Supplementary Figure 17. Closed-loop controller tracks the input independent of the absolute value of the output. (a) Simulated controller response in at different initial concentrations of P_X (0.1 - 4 nM) and the corresponding values of (b) $X/(\nu/\omega)$. To match the output levels, in the open-loop case initial P_Z^{tot} was both 0.1 nM while in the closed-loop case, P_Y^{tot} and P_Z^{tot} were both 1 nM each. The ODE model shown in Fig. 3b was used to determine the response with parameters shown in Table 1. The simulations were conducted for 8 hours, and the maximum value of $X(t)$ was used to determine the value of $X/(\nu/\omega)$. deGFP slopes were calculated from Z using (10) mentioned in Note 1.

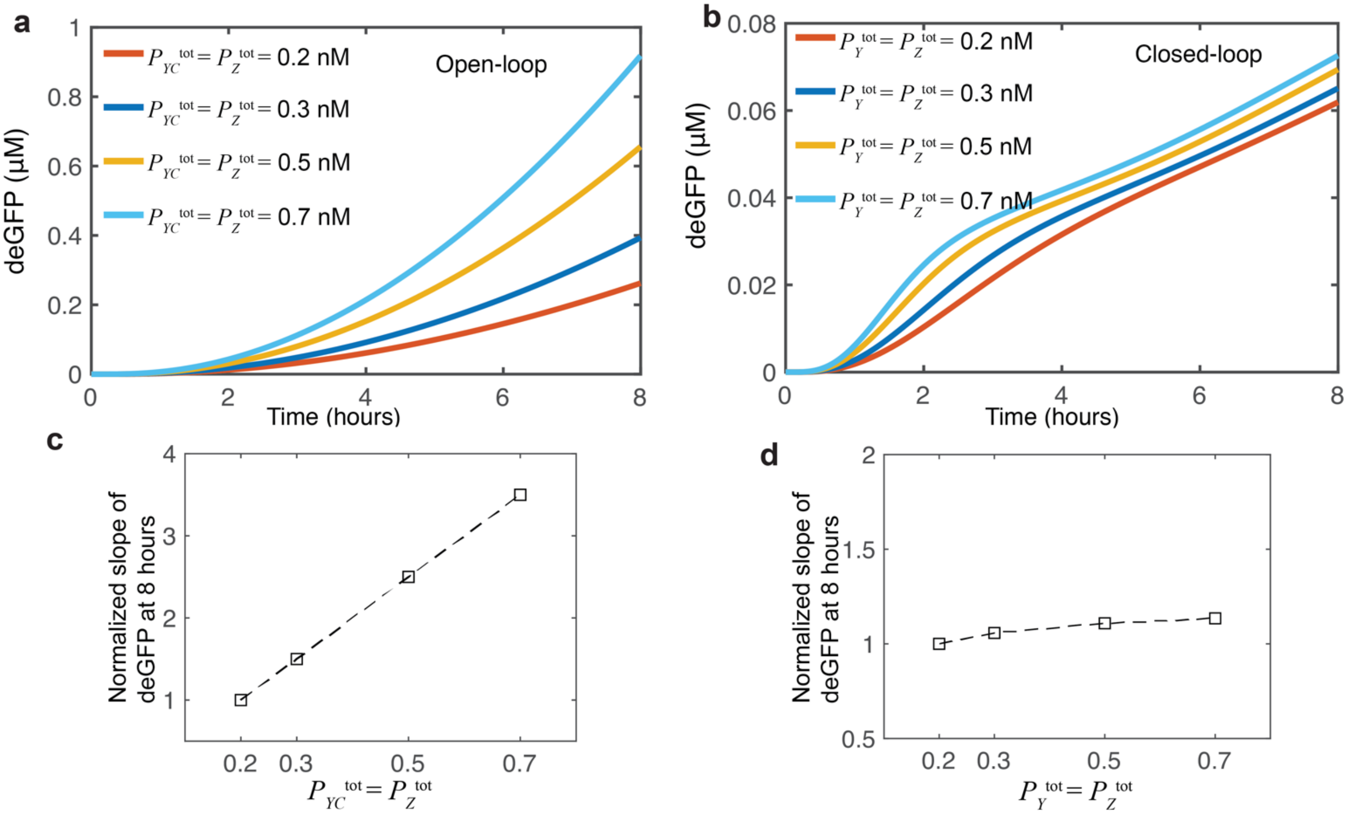

Supplementary Figure 18. Predicting the integral controller response in the presence of disturbances. Simulated controller response in the (a) open-loop and (b) close-loop cases when disturbances were introduced in the concentration of P_Y^{tot} and P_Z^{tot} and (c-d) the corresponding normalized change in the deGFP slopes at 8 hours respectively. The ODE model shown in Fig. 3b was used to determine the response with parameters shown in Table 1. Normalization was done with respect to the first slope value when P_Y^{tot} and P_Z^{tot}=0.2 nM.

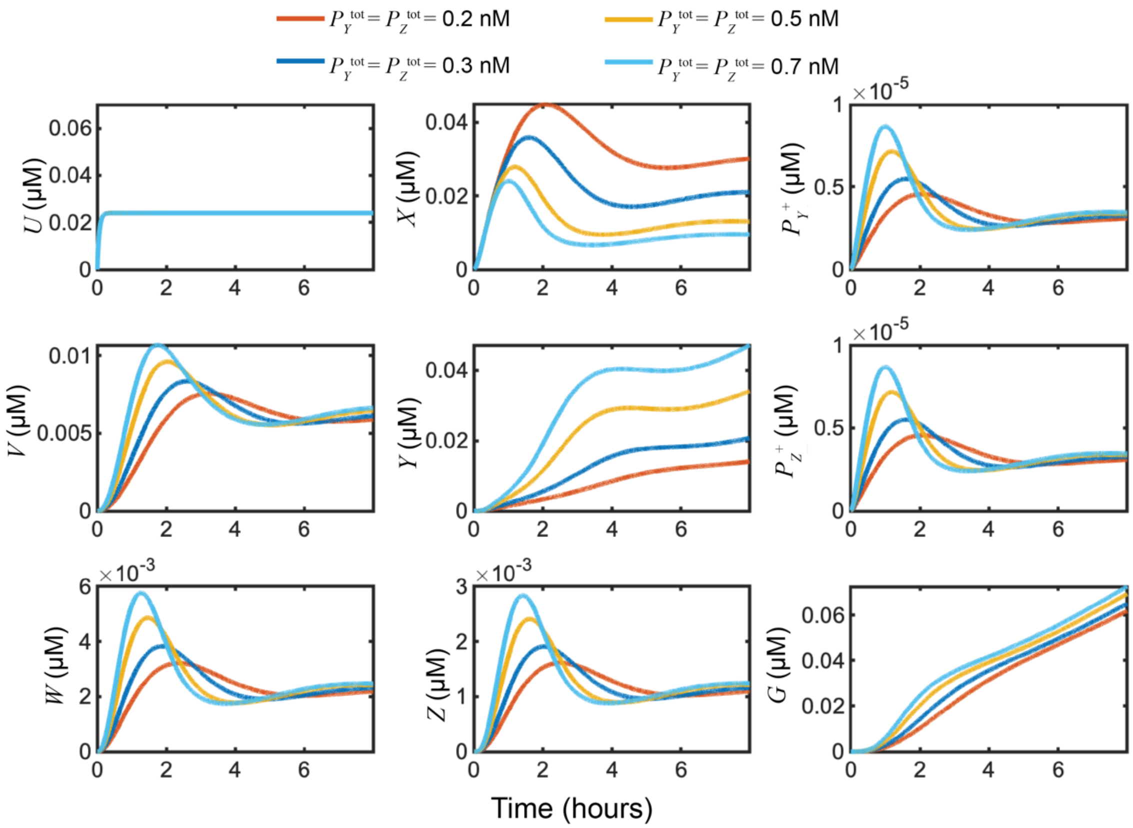

Supplementary Figure 19. Simulated closed-loop response of the controller to when disturbances were added in P_Y^{tot} and P_Z^{tot}. (a-b) Dynamic response of various species (see Fig. 3a) of the controller in the presence of disturbances in the concentration of P_Z^{tot} (0.2 – 0.7 nM) while initial P_X was 0.2 nM. The ODE model shown in Fig. 3b was used to determine the response with parameters shown in Table 1.

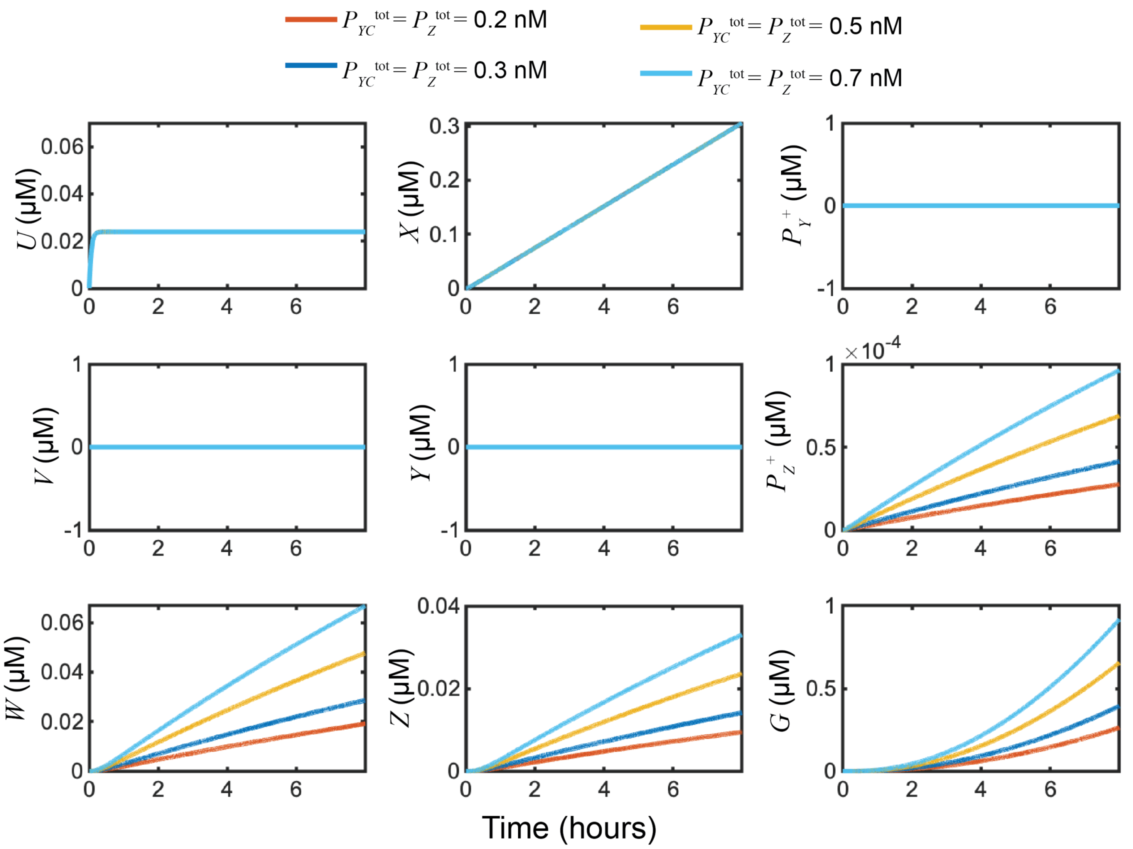

Supplementary Figure 20. Simulated open-loop response of the controller when disturbances were added in P_Z^{tot}. (a-b) Dynamic response of various species (Fig. 3a) of the controller in the presence of disturbances in the concentration of P_Z^{tot} (0.2 – 0.7 nM) while initial P_X was 0.2 nM. The ODE model shown in Fig. 3b was used to determine the response with parameters shown in Table 1.

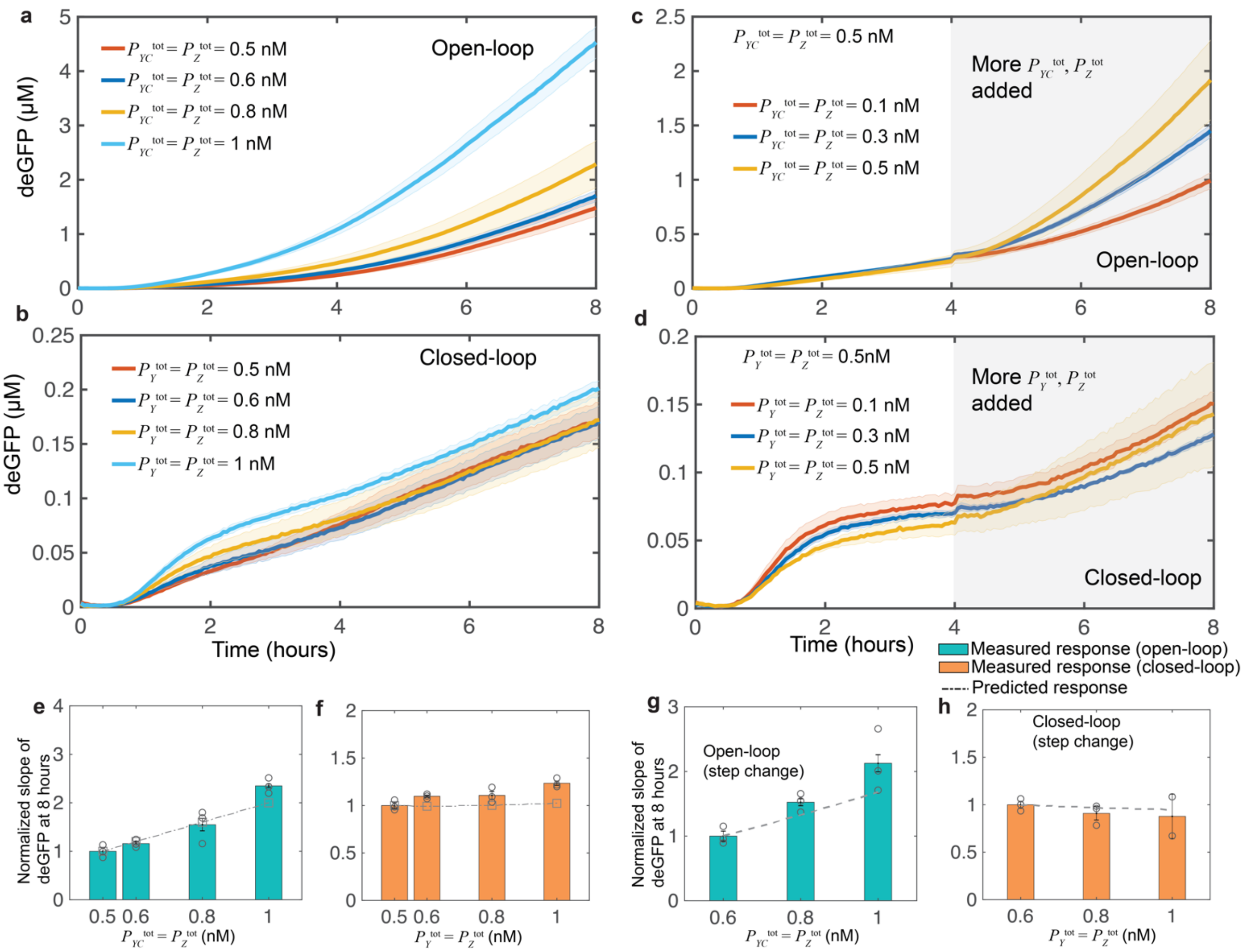

Supplementary Figure 21. Measuring the controller response to disturbances added in DNA concentrations at a different range. (a-b) TXTL measurement of the response of the integral controller in the presence of disturbances in the concentration of P_Y^{tot} and P_Z^{tot} (0.5 - 1 nM) for the (a) open-loop and (b) closed-loop cases while initial P_X was 0.5 nM. To disable the feedback in the open-loop case P_Y^{tot} was replaced by P_{YC}^{tot}, which expressed a protein that cannot sequester with X. (c-d) Measured response of the controller when the disturbance in P_Y^{tot} and P_Z^{tot} was added in a step manner. Additional P_Y^{tot} and P_Z^{tot} were added (0.1-0.5 nM) after 4 hours of the reaction in the presence of initial 0.5 nM of P_X, P_Y^{tot} and P_Z^{tot} each (see Methods). The error bars are shown in the shaded region and were determined using the standard error of the mean of two or more repeats. (e-h) Summary of the normalized deGFP slopes of the controller at 8 hours for (e, g) the open-loop and (f, h) closed-loop configurations. Normalization was done with respect to the first slope value for each variation in P_Y^{tot} and P_Z^{tot}. Before calculating deGFP slopes, measured deGFP responses were smoothed-out using the *rloess* smoothing method in MATLAB. The predicted response for each case was determined using the ODE model shown in Fig. 3b with parameters shown in Table 1. Source data are provided as a Source Data file.

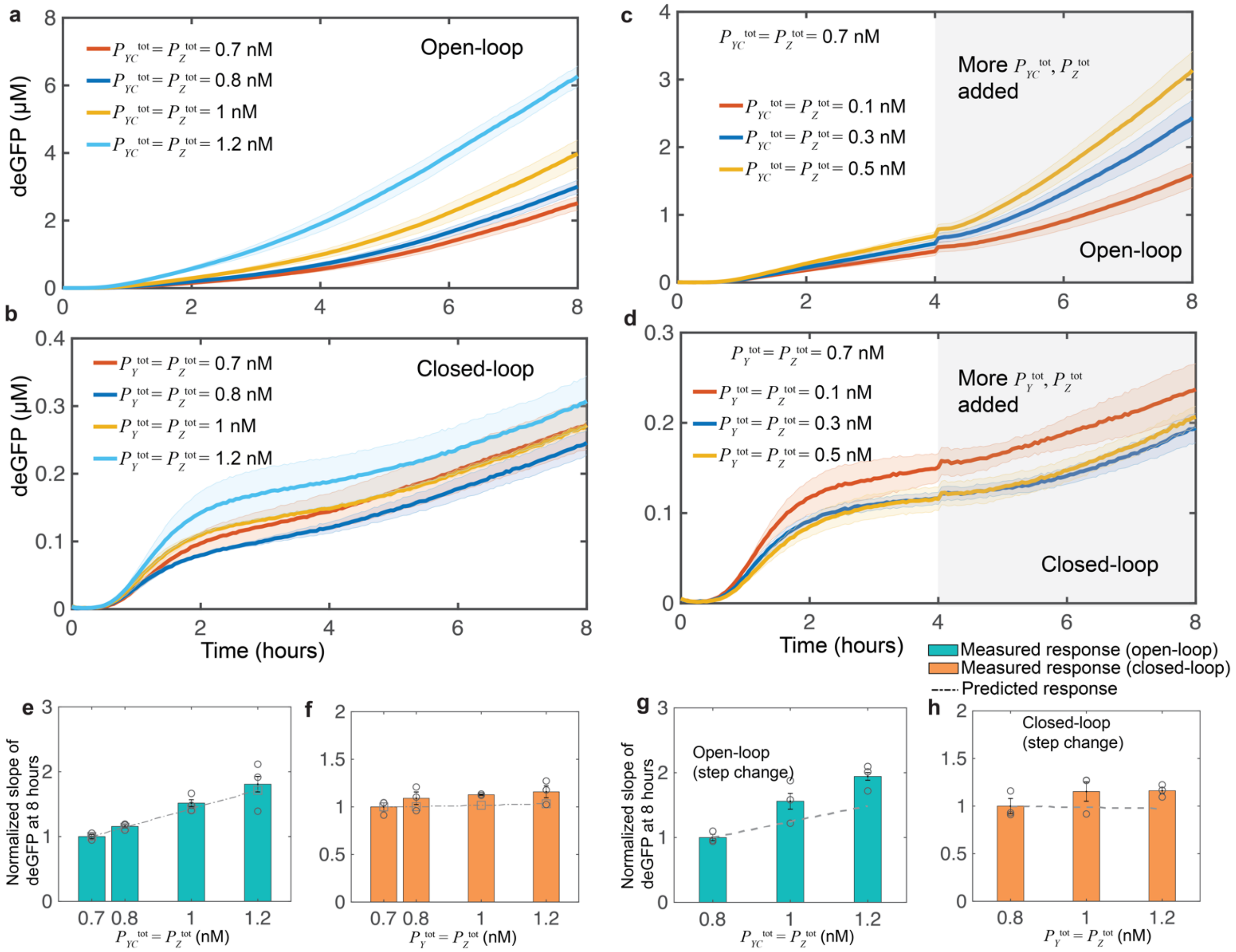

Supplementary Figure 22. Measuring the controller response to disturbances added in DNA at a different range. (a-b) TXTL measurement of the response of the integral controller in the presence of disturbances in the concentration of P_Y^{tot} and P_Z^{tot} (0.7 - 1.2 nM) for the (a) open-loop and (b) closed-loop cases while initial P_X was 0.7 nM. To disable the feedback in the open-loop case P_Y^{tot} was replaced by P_{YC}^{tot}, which expressed a protein that cannot sequester with *X*. (c-d) Measured response of the controller when the disturbance in P_Y^{tot} and P_Z^{tot} was added in a step manner. Additional P_Y^{tot} and P_Z^{tot} were added (0.1-0.5 nM) after 4 hours of the reaction in the presence of initial 0.7 nM of P_X, P_Y^{tot} and P_Z^{tot} each (see Methods). The error bars are shown in the shaded region and were determined using the standard error of the mean of two or more repeats. (e-h) Summary of the normalized deGFP slopes of the controller at 8 hours for (e, g) the open-loop and (f, h) closed-loop configurations. Normalization was done with respect to the first slope value for each variation in P_Y^{tot} and P_Z^{tot}. Before calculating deGFP slopes, measured deGFP responses were smoothed-out using the *rloess* smoothing method in MATLAB. The predicted response for each case was determined using the ODE model shown in Fig. 3b with parameters shown in Table 1. Source data are provided as a Source Data file.

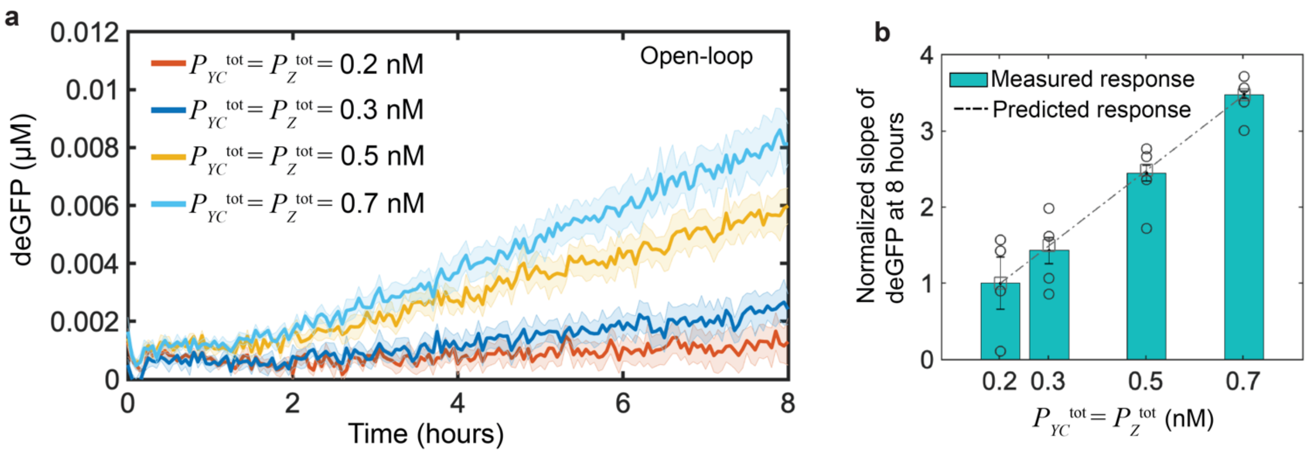

Supplementary Figure 23. Open-loop controller cannot suppress the disturbances independent of the absolute value of the output. (a) TXTL measurement of the response of the integral controller in the presence of disturbances in the concentration of P_{YC}^{tot} and P_Z^{tot} (0.2 - 0.7 nM) for the open-loop case while initial P_X was 0.02 nM. Error bars are shown in the shaded region and were determined using the standard error of the mean of three or more repeats. (b) Summary of the normalized deGFP slopes of the controller at 8 hours. Normalization was done with respect to the first slope value. Before calculating deGFP slopes, measured deGFP responses were smoothed-out using the *rloess* smoothing method in MATLAB. The predicted response for each case was determined using the ODE model shown in Fig. 3b with parameters shown in Table 1. Source data are provided as a Source Data file.

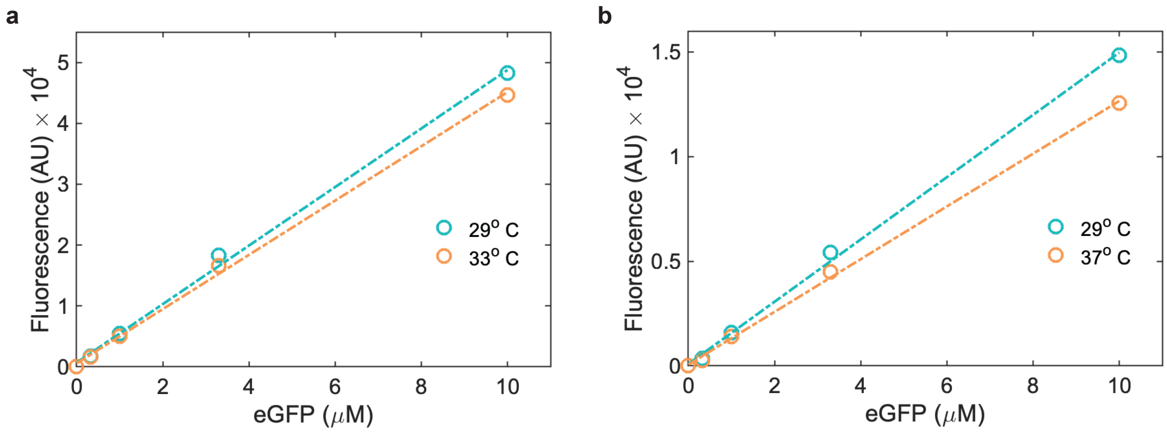

Supplementary Figure 24. Standard curve of GFP at 29° C and 33° C, and 29° C and 37° C. A two-degree polynomial was used to fit the endpoints and the corresponding R-square value is 0.99 for all the cases. The slopes at (a) 33° C and (b) 37° C are reduced only by factors of (a) 1.07 and (b) 1.18 compared to 29° C respectively. To account this effect, all the data collected at 33° C and 37° C were multiplied by the respective factors to get the accurate, temperature-adjusted, GFP concentration.

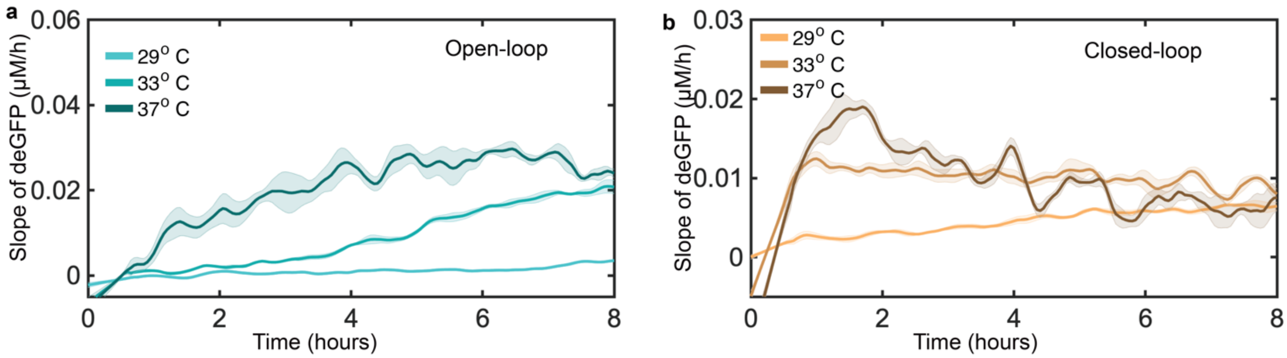

Supplementary Figure 25. The corresponding deGFP slopes for the date shown in Fig. 6 (main text) for the (a) open-loop (P_{YC}^{tot} and P_Z^{tot} were both 0.1 nM) and (b) closed-loop (P_Y^{tot} and P_Z^{tot} were both 1 nM each) cases while initial P_X was 0.1 nM. Before calculating deGFP slopes, measured deGFP responses were smoothed-out using the *rloess* smoothing method in MATLAB. Source data are provided as a Source Data file.

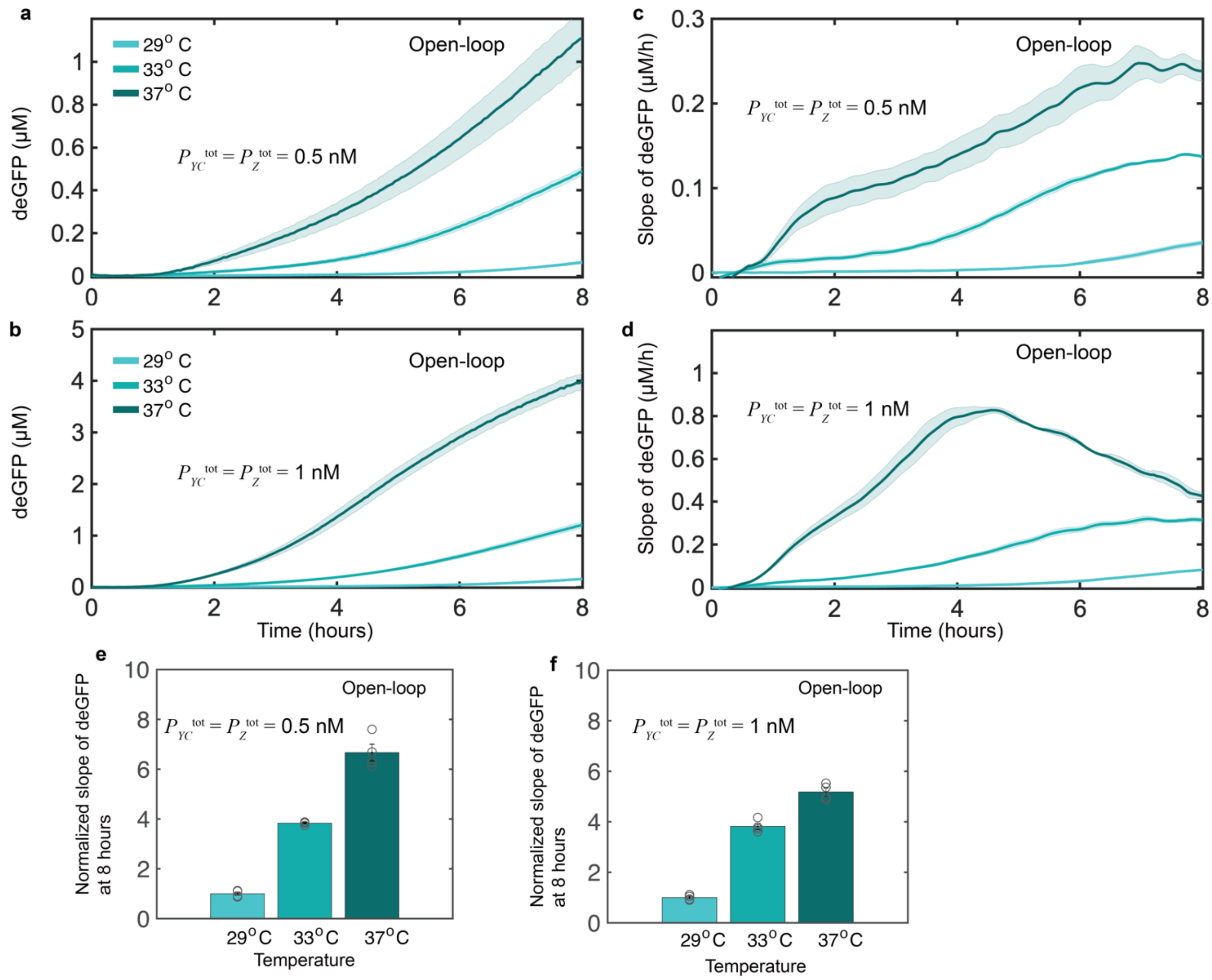

Supplementary Figure 26. Open-loop controller cannot suppress the global disturbance independent of the absolute value of the output. (a,b) Measured response of the controller at three different external constant change in the reaction temperatures for the open-loop case while initial P_X was 0.1 nM, and (a) P_{YC}^{tot} and P_Z^{tot} were both 0.5 nM and (b) P_{YC}^{tot} and P_Z^{tot} were both 1 nM each. (c,d) The corresponding deGFP slopes and (e,f) summary of the normalized deGFP slopes at 8 hours respectively. The error bars are shown in the shaded region and were determined using the standard error of the mean of two or more repeats. The responses shown in (c, d) were normalized with respect to the deGFP slope value recorded at 29° C. Plate readers were calibrated at 29° C, 33° C and 37° C separately to a standard curve of GFP to ensure fluorescence variation reflects protein concentration variation (see **Supplementary** Fig. 24) while at 33° C, the same standard curve of GFP was used as for 29° C. Before calculating deGFP slopes, measured deGFP responses were smoothed-out using the *rloess* smoothing method in MATLAB. Source data are provided as a Source Data file.

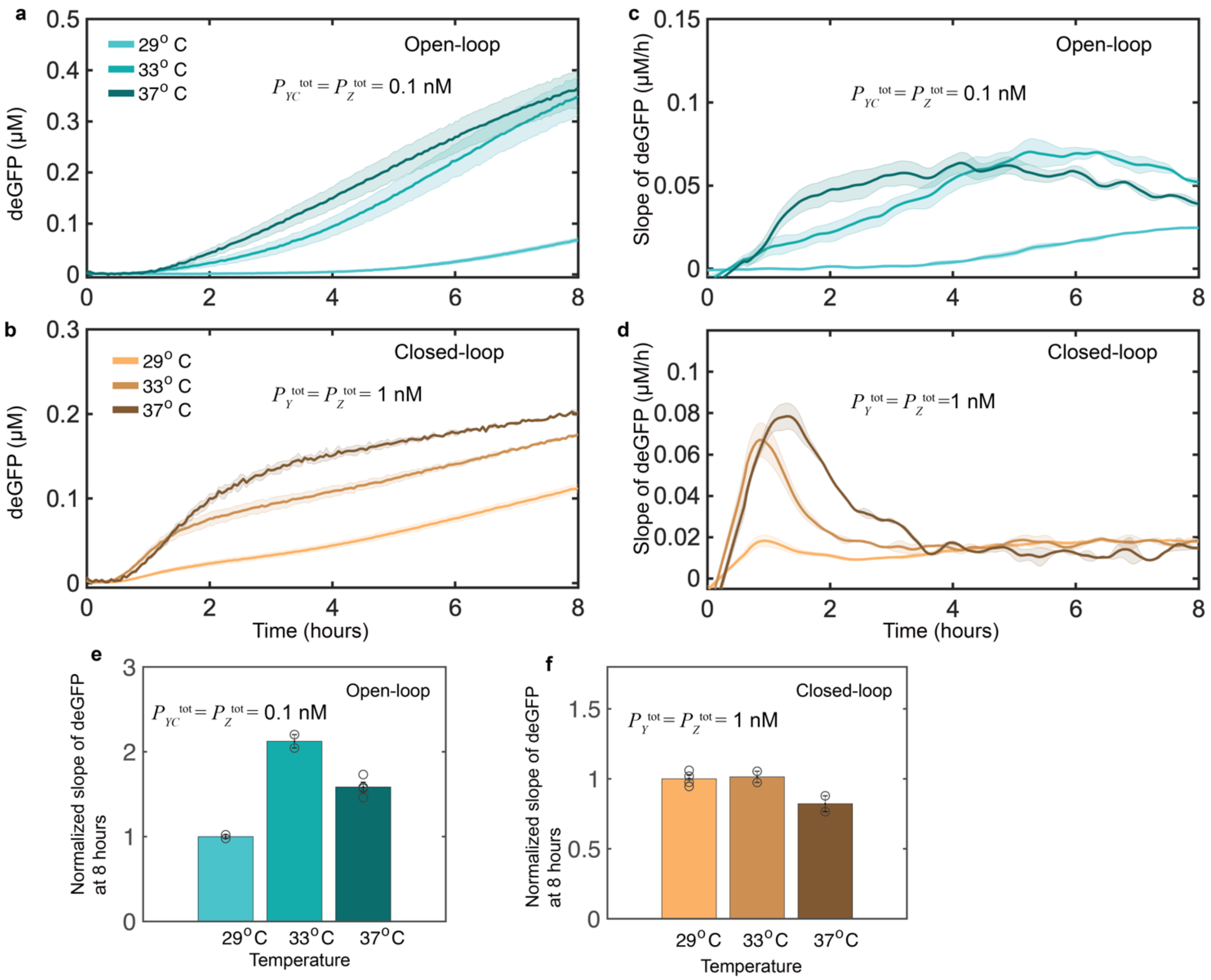

Supplementary Figure 27. Closed-loop controller suppresses global disturbance at a different value of the input P_X. (a,b) Measured response of the controller at three different external constant change in the reaction temperatures for the (a) open-loop (P_{YC}^{tot} and P_Z^{tot} were both 0.1 nM) and (b) closed-loop (P_Y^{tot} and P_Z^{tot} were both 1 nM each) cases while initial P_X was 0.3 nM. (c,d) The corresponding deGFP slopes and (e,f) summary of the normalized deGFP slopes at 8 hours respectively. To disable the feedback in the open-loop case, P_Y^{tot} was replaced by P_{YC}^{tot}, which expresses a protein that cannot sequester with X. The error bars are shown in the shaded region and were determined using the standard error of the mean of two or more repeats. The responses shown in (c, d) were normalized with respect to the deGFP slope value recorded at 29° C. Plate readers were calibrated at 29° C, 33° C and 37° C separately to a standard curve of GFP to ensure fluorescence variation reflects protein concentration variation (see **Supplementary** Fig. 24) while at 33° C, the same standard curve of GFP was used as for 29° C. The closed-loop behavior is unchanged (within experimental error), while the open-loop behavior changes drastically. Before calculating deGFP slopes, measured deGFP responses were smoothed-out using the *rloess* smoothing method in MATLAB. Source data are provided as a Source Data file.

Supplementary Note 2

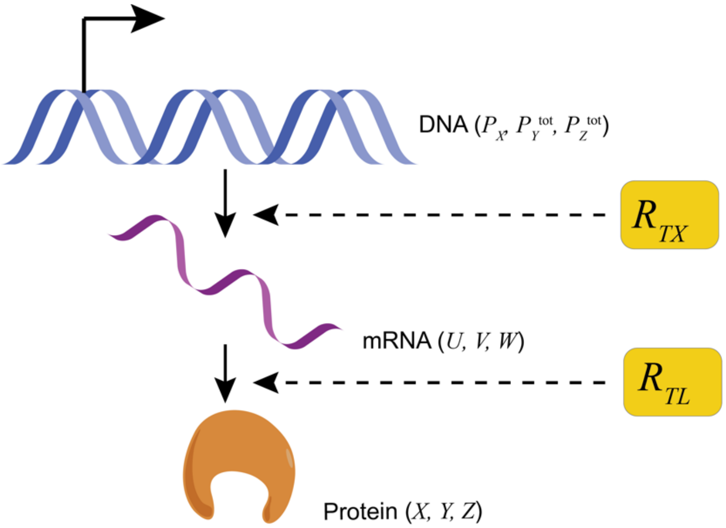

Supplementary Figure 28. Schematic representation of the framework used to model the depletion of energy resources in the TXTL reactions. Here, R_{TX} and R_{TL} represent the initial constant resources available in the reaction mixture that facilitate transcriptional and translation reactions respectively. The production rate of mRNA will be DNA concentration-dependent including steady-state level and saturation effect, and the production rate of protein will be mRNA concentration-dependent which governs the consumption rate of R_{TL}.[2]

The updated ODE model of the controller to account for the resource competition and depletion in measured responses:

$$\frac{dU}{dt} = \alpha_U \frac{R_{TX} P_X}{K_{TX} + P_X} - \delta_U U, \tag{53}$$

$$\frac{dX}{dt} = \beta_X \frac{R_{TL} U}{K_1 + U} - \kappa XY + \kappa_i [XY] - \omega X P_Y + \nu P_Y^+ - \omega X P_Z + \nu P_Z^+, \tag{54}$$

$$\frac{dP_Y^+}{dt} = \omega X P_Y - \nu P_Y^+, \tag{55}$$

$$\frac{dV}{dt} = \alpha_V \frac{R_{TX} P_Y}{K_{TX} + P_Y} + \alpha_V^+ \frac{R_{TX} P_Y^+}{K_{TX} + P_Y^+} - \delta_V V, \tag{56}$$

$$\frac{dY}{dt} = \beta_Y \frac{R_{TL} V}{K_1 + V} - \kappa XY + \kappa_i [XY], \tag{57}$$

$$\frac{dW}{dt} = \alpha_W \frac{R_{TX} P_Z}{K_{TX} + P_Z} + \alpha_W^+ \frac{R_{TX} P_Z^+}{K_{TX} + P_Z^+} - \delta_W W, \tag{58}$$

$$\frac{dP_Z^+}{dt} = \omega X P_Z - \nu P_Z^+, \tag{59}$$

$$\frac{dZ}{dt} = \beta_Z \frac{R_{TL} W}{K_1 + W} - \gamma_G Z, \tag{60}$$

$$\frac{dG}{dt} = \gamma_G Z, \tag{61}$$

$$\begin{aligned} \frac{dR_{TX}}{dt} &= \delta_{TX} \frac{R_{TX} P_X}{K_{TX} + P_X} - \delta_{TX} \frac{R_{TX} P_Y}{K_{TX} + P_Y} .. \\ &- \delta_{TX} \frac{R_{TX} P_Y^+}{K_{TX} + P_Y^+} - \delta_{TX} \frac{R_{TX} P_Z}{K_{TX} + P_Z} - \delta_{TX} \frac{R_{TX} P_Z^+}{K_{TX} + P_Z^+}, \end{aligned} \tag{62}$$

$$\frac{dR_{TL}}{dt} = -\delta_{TL} \frac{R_{TL} U}{K_{TL} + U} - \delta_{TL} \frac{R_{TL} V}{K_{TL} + V} - \delta_{TL} \frac{R_{TL} W}{K_{TL} + W}. \tag{63}$$

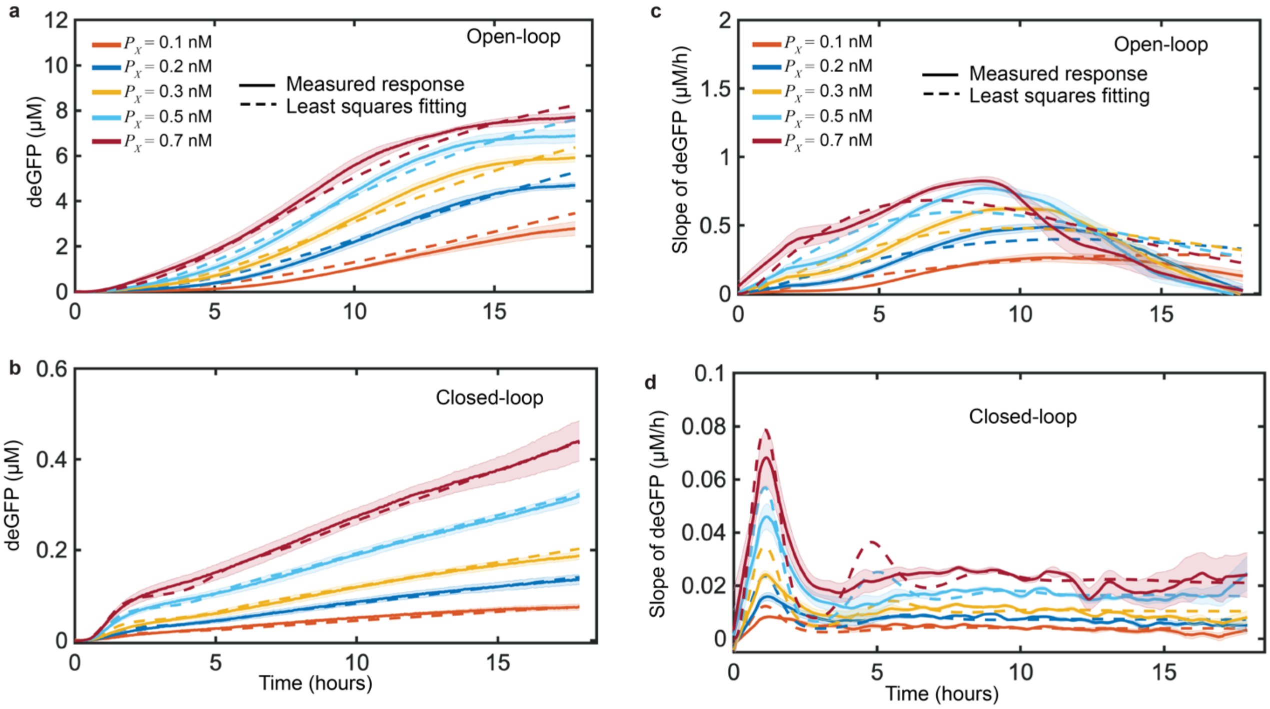

Supplementary Figure 29: **Updated ODE model is capable of following the effect of resource limitation in the TXTL reactions.** (a-b) Comparing the updated model response with the measured deGFP response of the integral controller in the (a) open-loop and (b) closed-loop configurations at different initial concentrations of P_X (0.1 - 0.7 nM) while initial P_Y^{tot} and P_Z^{tot} were both 1 nM. (c-d) Corresponding deGFP slopes for the (c) open-loop and (d) closed-loop operations. To disable the feedback in the open-loop case, P_Y^{tot} was replaced by P_{YC}^{tot}. Error bars are from the SEM of at least three repeats. The ODE model shown in (53)-(63) was used to determine the response with parameters shown in Table 1 and other parameters were $K_{TX} = 4.23\times10^{-7}$ M, $\delta_{TX} = 9.08\times10^{-6}$ s^{-1}, $K_{TL} = 1.13\times10^{-5}$ M, $\delta_{TL} = 0.00066$ s^{-1}, $K_I = 3.31\times10^{-5}$ M, $R_{TX} = 1.99\times10^{-7}$, $R_{TL} = 8.06\times10^{-5}$. Unlike the rest of the cases, while modeling the open-loop response, only κ and κ_i were set to zero. This is to account for the TXTL resource consumption by *yc* gene (control gene for open-loop case). Before calculating deGFP slopes, measured deGFP responses were smoothed-out using the *rloess* smoothing method in MATLAB. Source data are provided as a Source Data file.

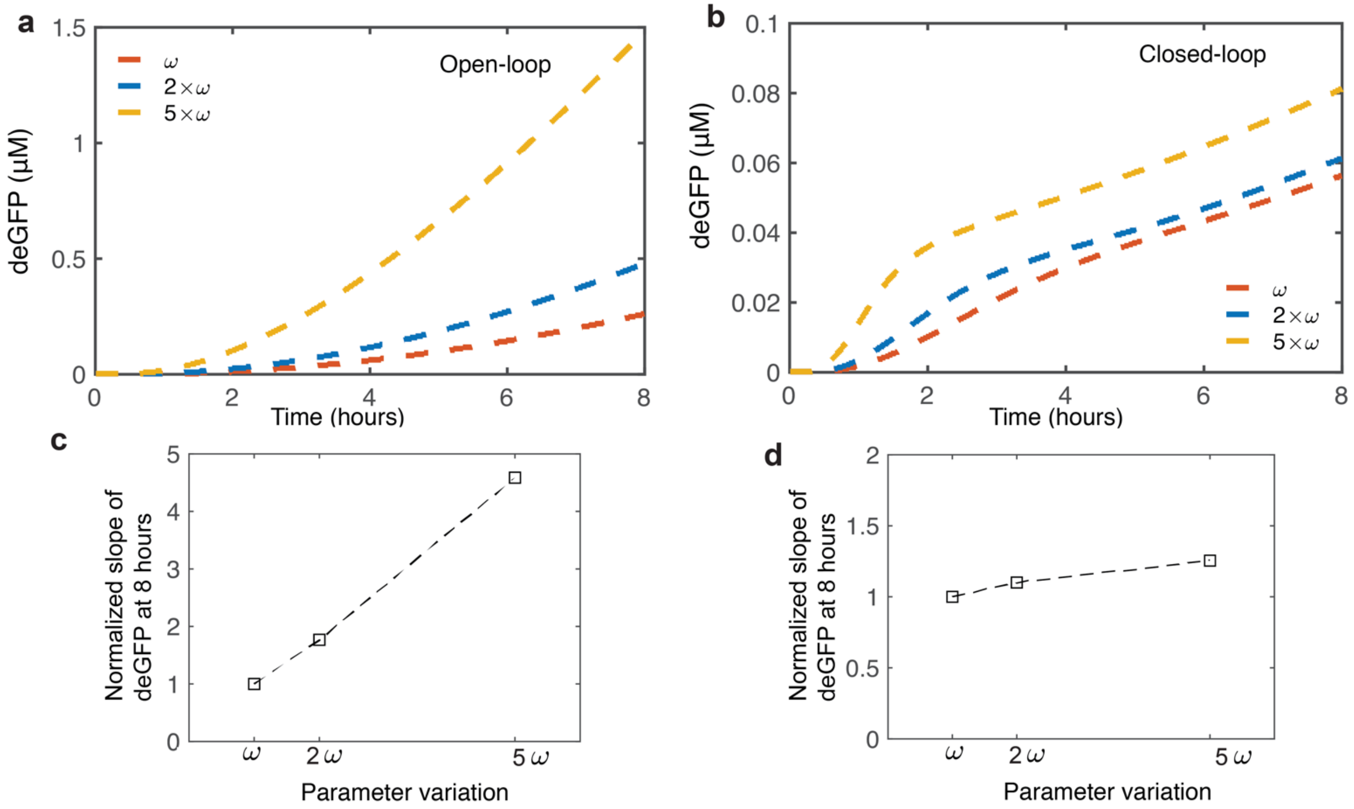

Supplementary Figure 30. Closed-loop controller can suppress the disturbances added in the association rate *ω*. Simulated controller response in the (a) open-loop (P_Z^{tot} = 1 nM) and (b) close-loop (P_Y^{tot} = P_Z^{tot} = 1 nM) cases when disturbances were introduced in *ω* and (c-d) normalized change in the deGFP slopes at 8 hours respectively. Initial P_X was 0.02. The ODE model shown in Fig. 3b was used to determine the response with parameters shown in Table 1. Normalization was done with respect to the deGFP slope calculated at the nominal value of *ω*.

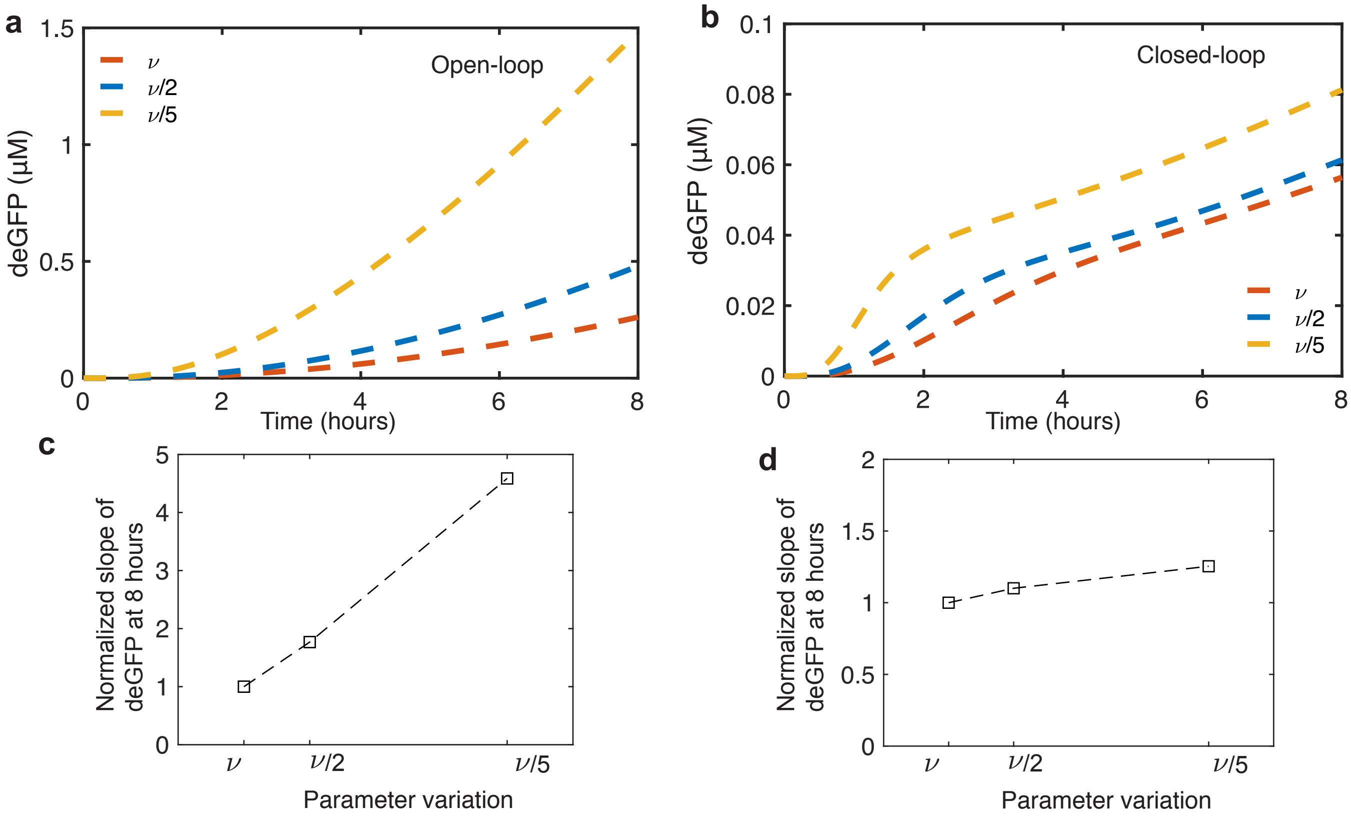

Supplementary Figure 31. Closed-loop controller can suppress the disturbances added in the dissociation rate ν. Simulated controller response in the (a) open-loop (P_Z^{tot} = 1 nM) and (b) close-loop ($P_Y^{tot} = P_Z^{tot}$ = 1 nM) cases when disturbances were introduced in ν and (c-d) normalized change in the deGFP slopes at 8 hours respectively. Initial P_X was 0.02 nM. The ODE model shown in Fig. 3b was used to determine the response with parameters shown in Table 1. Normalization was done with respect to the deGFP slope calculated at the nominal value of ν.

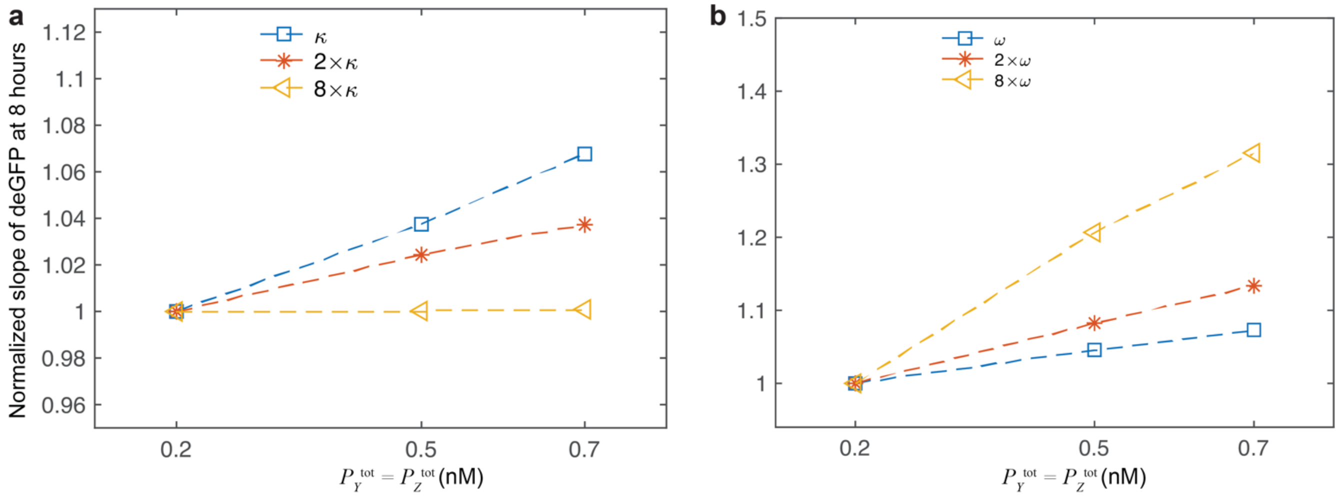

Supplementary Figure 32. Effect of the disturbance on the closed-loop output as a function of sequestration and activation association rates. Simulated closed-loop controller response for different values of the (a) sequestration rate (κ) and (b) activation association rate (ω). Initial P_X was 0.02 nM while initial P_Y^{tot} and P_Z^{tot} were both 1 nM each. The ODE model shown in Fig. 3b was used to determine the response with parameters shown in Table 1, and the values are reported at 12 hours. Normalization was done with respect to the deGFP slopes calculated at the nominal parameter values.

Supplementary Table 1. List of constraints placed on the parameters during the fitting. These constraints are in agreement with the published literature, and allowed us to determine the parameter values that are biologically realistic.[3-6]

Parameters	Minimum Value	Maximum Value	Units
α_U	0.01	0.8	s^{-1}
α_V	10^{-8}	10^{-4}	s^{-1}
α_V^+	0.01	0.8	s^{-1}
α_W	10^{-8}	10^{-4}	s^{-1}
α_W^+	0.01	0.8	s^{-1}
δ_U	10^{-5}	10^{-2}	s^{-1}
δ_V	10^{-5}	10^{-2}	s^{-1}
δ_W	10^{-5}	10^{-2}	s^{-1}
β_X	10^{-4}	10^{-2}	s^{-1}
β_Y	10^{-4}	10^{-2}	s^{-1}
β_Z	10^{-4}	10^{-2}	s^{-1}
κ	10^{4}	10^{7}	$M^{-1}\ s^{-1}$
κ_i	10^{-5}	10^{-3}	s^{-1}
ω	10^{4}	10^{6}	$M^{-1}\ s^{-1}$
ν	10^{-2}	10	s^{-1}
Υ_G	10^{-4}	10^{-2}	s^{-1}

References

1. Ogata, K. & Yang, Y. *Modern control engineering*. Vol. 4 (Prentice-Hall, 2002).
2. Stogbauer, T.; Windhager, L.; Zimmer, R.; Radler, J. O., Experiment and mathematical modeling of gene expression dynamics in a cell-free system. *Integr Biol (Camb)* **2012,** *4* (5), 494-501.
3. Siegal-Gaskins, D., Tuza, Z. A., Kim, J., Noireaux, V. & Murray, R. M. Gene circuit performance characterization and resource usage in a cell-free "breadboard". *ACS Synth Biol* **3**, 416-425, doi:10.1021/sb400203p (2014)
4. Garamella, J., Marshall, R., Rustad, M. & Noireaux, V. The All E-coli TX-TL Toolbox 2.0: A Platform for Cell-Free Synthetic Biology. *Acs Synthetic Biology* **5**, 344-355, doi:10.1021/acssynbio.5b00296 (2016).
5. Hu, C. Y., Varner, J. D. & Lucks, J. B. Generating Effective Models and Parameters for RNA Genetic Circuits. *ACS Synth Biol* **4**, 914-926, doi:10.1021/acssynbio.5b00077 (2015).
6. Westbrook, A. M. & Lucks, J. B. Achieving large dynamic range control of gene expression with a compact RNA transcription-translation regulator. *Nucleic Acids Res* **45**, 5614-5624, doi:10.1093/nar/gkx215 (2017).
